# The ESMO-Magnitude of Clinical Benefit Scale (ESMO-MCBS) visualisation: picturing the evidence of clinical benefit of clinical trial data

**DOI:** 10.1016/j.esmorw.2025.100171

**Published:** 2025-08-26

**Authors:** C.G. Urzua-Traslavina, N.J. Latino, M. Galotti, N.I. Cherny, S.F. Oosting, E.G.E. de Vries, R.S.N. Fehrmann

**Affiliations:** 1 Department of Medical Oncology, University Medical Center Groningen, University of Groningen, Groningen, The Netherlands; 2 Oncode Institute, Utrecht, The Netherlands; 3 European Society for Medical Oncology (ESMO) Head Office, ESMO, Lugano, Switzerland; 4 Department of Medical Oncology, Cancer Pain and Palliative Medicine Service, Shaare Zedek Medical Center, Jerusalem, Israel

**Keywords:** ESMO-MCBS, visualisation, anticancer treatment, clinical benefit

## Abstract

**Background:**

Choosing the right cancer treatment is crucial for patients and health care professionals, while health care systems must allocate resources efficiently. The European Society for Medical Oncology-Magnitude of Clinical Benefit Scale (ESMO-MCBS) assists decision making by assessing the clinical benefit of anticancer medications. ESMO developed and field-tested a visualisation of the ESMO-MCBS scores to increase its user-friendliness and effectiveness.

**Methods:**

An expert panel iteratively designed and internally reviewed a prototype of the visualisation, then refined it through field-testing. A survey was conducted concurrently with the field-testing to evaluate the understandability of the visualisation. Field-testing materials and survey questions were made accessible to the public on the ESMO website and promoted at the ESMO Congress 2019 through ESMO social media platforms. The insights gathered from all the feedback were instrumental in refining the design. Subsequently, a conclusive survey was deployed during the ESMO Congress 2023 to assess the final version of the visualisation.

**Results:**

A two-panel visualisation was developed to reflect the steps of the ESMO-MCBS scoring procedure. Field-testing included visualisations for 121 studies, each based on their ESMO-MCBS scores. Feedback from the survey (*n* = 56 experts) indicated that oncology-related professions and statisticians, as well as respondents with previous knowledge of the ESMO-MCBS, showed a sufficient understanding of the visualisations while indicating areas to improve. Incorporating the survey’s feedback, the revised visualisations were then integrated into the publicly available ESMO-MCBS Scorecards, and a final survey (*n* = 118 experts) was conducted to evaluate these revisions. More than 80% of the respondents reported the visualisation to be accessible and effective at communicating the score.

**Conclusions:**

The visualisations offer a visual understanding of the ESMO-MCBS scores by enhancing user-friendliness and understandability.

## Highlights

• Choosing the right cancer drug is vital for patients, doctors, and regulators.
• Visualisation aids decisions and boosts transparency and accountability.
• An ESMO-MCBS visual tool was tested and refined for broad understanding.
• All ESMO-MCBS visuals and scorecards are available on the ESMO website.

## Background

An increasing number of high-priced medicines are available for the treatment of patients with cancer.^1^ However, their cost is often not related to the magnitude of clinical benefit delivered.^2^, ^3^, ^4^ This discrepancy has motivated a growing emphasis on clinical benefit to frame the appropriate use of limited resources and optimise the development of effective and affordable cancer care.^5^

Developed by the European Society for Medical Oncology (ESMO), the ESMO-Magnitude of Clinical Benefit Scale (ESMO-MCBS) offers a validated scoring system to categorise the anticipated clinical benefit of anticancer therapies.^6^^,^^7^ This tool supports clinicians, policymakers, and patients to make informed decisions regarding treatment selection and resource allocation. The ESMO-MCBS leverages a comprehensive set of criteria, including overall survival, progression-free survival, disease-free survival, hazard ratio, response rate, quality of life, underlying disease prognosis, and treatment toxicity, to generate a score reflecting the anticipated clinical benefit.

The ESMO-MCBS is internationally recognised as a valuable tool to assess the value of cancer medicines.^8^, ^9^, ^10^ Given that patients, health care professionals, and regulatory agencies each have unique considerations when evaluating anticancer treatments,^5^ understanding the factors that drive the ESMO-MCBS scores is crucial. Such clarity can tailor the decision-making process to the specific needs of different stakeholders.

The human brain excels at processing visual information. Visualisations leverage this strength, allowing users to identify patterns and trends in the data that might otherwise be overlooked or not comprehended.^11^ Data visualisations vary widely, from basic graphs to sophisticated interactive displays that are crafted to simplify complex data for easier interpretation.^12^ Visualisations translate data into a more intuitive format, making it easier to grasp relationships, patterns, and trends within the data,^11^ even to those with limited health literacy or low language proficiency.^13^ Furthermore, visual information is generally better retained than raw numbers, creating a stronger cognitive association with the data, leading to improved memory and recall of key information.^14^

Design is a critical issue, since poorly designed visualisation may contribute to risk of misinterpretation that would counter the original intent.^15^ To mitigate misinformation, involving the target audience in the design process and verifying comprehension is an important part of the development process for visualisations.^13^

ESMO has developed a visualisation of the ESMO-MCBS scores to illustrate the factors contributing to the final score. The visualisation aimed to consolidate all the different ESMO-MCBS evaluation forms into a unified graphical interface, increasing its user-friendliness and understandability. This manuscript details the development, field-testing, and validation of the ESMO-MCBS visualisation.

## Methods

### Development of the prototype ESMO-MCBS visualisation

The project began in 2018, marking a collaborative effort between ESMO and the University Medical Center Groningen (UMCG). After multiple discussion meetings, a tentative design was conceived in January 2019, and Netgen, a Swiss agency specialising in user experience and web development,^16^ was contacted to implement and develop the visualisation.

From May to September 2019, the tentative design underwent iterative development, enriched by continuous input from ESMO, UMCG, and Netgen representatives. An advanced design showcasing visualisations for five treatments scored with ESMO-MCBS v1.1 was internally evaluated by the ESMO-MCBS Working Group (Supplementary File S1, available at https://doi.org/10.1016/j.esmorw.2025.100171). The insights gained from this review shaped the first prototype, which was then expanded to cover 121 trials scored using ESMO-MCBS v1.1 and used for the open field-testing phase.

### Survey implementation

To assess the visualisation prototype's user-friendliness and comprehensibility to a broad audience, ESMO conducted a survey using the online platform Qualtrics.^17^ From September 2019 to January 2020, a public link was made available on the ESMO website. The link included the survey and a brief tutorial of the visualisations (Supplementary Files S2 and S3 respectively, available at https://doi.org/10.1016/j.esmorw.2025.100171), and a catalogue showcasing 121 ESMO-MCBS v1.1 scored trials, including 12 with curative and 109 non-curative intent. The survey was disseminated through ESMO’s social media and actively promoted at the ESMO Congress 2019.

The survey was divided into five sections. Section 1 (questions 1-6) gathered data on respondents’ backgrounds and previous familiarity with the ESMO-MCBS. Section 2 (questions 7-14) addressed the visualisation layout, prompting users to identify and evaluate elements such as the preliminary and final scores, adjustment factors, pending data, and quality-of-life (QoL) improvements. Section 3 (questions 15-22) focussed on interpreting the icons used within the visualisation. Section 4 (questions 23-26) solicited respondents' preferences between two visualisations with identical final scores, aiming to gather subjective appeal.

## Results

### The ESMO-MCBS v1.1 visualisation

The ESMO-MCBS visualisation, designed to reflect the scoring process of ESMO-MCBS v1.1,^17^^,^^18^ support two formats for each treatment, namely a ‘detailed’ version with two panels and a ‘summarised’, smaller version.

**Figure 1:**
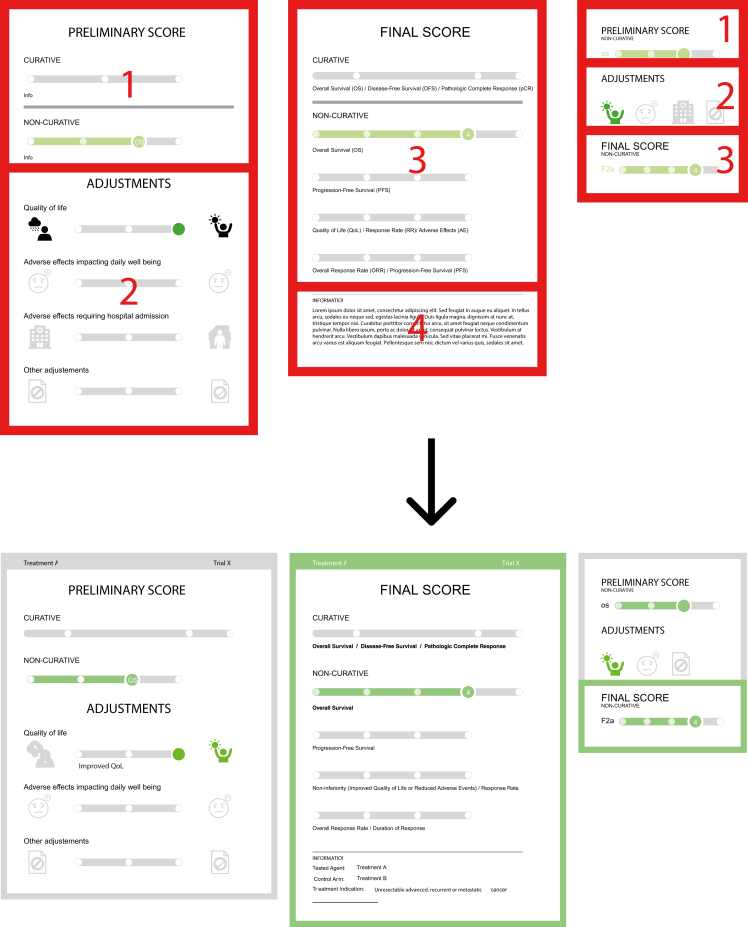
**Overview of prototype and final version of the visualisation.** Upper panel: prototype version of the visualisation with highlighted sections. Lower panel: final version visualisation of the same treatment.

In the detailed version (Figure 1, left), the left panel displays the preliminary score at the top (1) and the adjustments at the bottom (2), while the right panel shows the final score (3) at the top and an information box (4) at the bottom. The summarised version presents the equivalent sections of the preliminary score, adjustments, and final score in three consecutive sections in one panel (Figure 1, right).

Visualisations are colour-coded based on the colour of the form used to score the treatment: blue for form 1, green for 2a, terracotta for 2b, purple for 2c, and grey for form 3.^19^

Adjustments are classified into three categories: QoL, serious and disabling adverse effects, and other adjustments such as study crossover and data maturity (Table 1). The positioning of the adjustments on the left, right, or centre indicates whether the benefit for the patient is worse, better, or equivalent compared with the control arm. Green or red coloured adjustment icons signify an upgrade or downgrade of the score respectively. In situations when there was no QoL assessment or if it was only exploratory, it is not depicted (Table 2).

**Table 1:** Types of adjustments in ESMO-MCBS v1.1 that can influence the ESMO-MCBS score

Adjustments	Upgrade	Downgrade
QoL	Forms 2a, b, 3	Form 2b
Grade 3-4 toxicities impacting daily well-being	Forms 2a, b, 3	Form 3
Presence of incremental toxicity		Form 2b
Long-term plateau in survival curve	Form 2a	
Long-term plateau in the PFS curve	Form 2b	
Early crossover	Form 2b	
Confirmatory phase IV experience	Form 3	
Only improved PFS mature data shows no OS advantage and no improved QoL		Form 2b
Improvement also observed in OS	Form 2b	

ESMO-MCBS, ESMO-Magnitude of Clinical Benefit Scale; OS, overall survival; PFS, progression-free survival; QoL, quality of life.

**Table 2:** Translation of form modifiers to visualisation icons

Symbols			
QoL adjustments	Other adjustments^a^	Serious and disabling adverse effects	Notes
			**Affected score** Red = negative/deterioration Green = positive/improvement
			**No data/no indications**
			**Pending data**
			**No QoL benefit shown**

**Slider dots**			

**Dot image**	**Colour**	**Meaning**	

	Red	Negative	
	Green	Positive	
	Grey	Neutral	
	Grey +?	Neutral with pending data	

a Other adjustments include: long-term plateau in the survival curve, long-term plateau in the progression-free survival (PFS) curve, early stopping or crossover, only improved PFS mature data shows no overall survival advantage and no improved quality of life (QoL).

When pending data for a treatment can modify the ESMO-MCBS score, the slider is placed in the middle with a question mark to indicate potential future score changes (i.e. this is illustrated in the visualisation, namely when QoL is an endpoint with pending results).

### Survey of ESMO-MCBS visualisation: results

From August to October 2019, ESMO collected 54 responses, with 10 being partial, from professionals across 24 countries, predominantly oncologists.

#### Familiarity with ESMO-MCBS (section 1)

Of the respondents, 88% were familiar with ESMO-MCBS, primarily through ‘international events/congresses’ and by ‘word of mouth’.

#### Comprehension of the visualisations (sections 2 and 3)

Statisticians and oncology professionals (reported professions: ‘Statistician, ‘Biostatistician’, ‘Oncologist’, ‘Fellow medical oncology’, ‘Oncology pharmacist’, ‘Pharma representative’) demonstrated the highest accuracy in understanding the visualisations (Supplementary Figure S1, available at https://doi.org/10.1016/j.esmorw.2025.100171). Those without prior knowledge of ESMO-MCBS had a lower correct response rate (72%) compared with those with prior knowledge (89%).

#### Preference for visualisation example (section 4)

**Figure 2:**
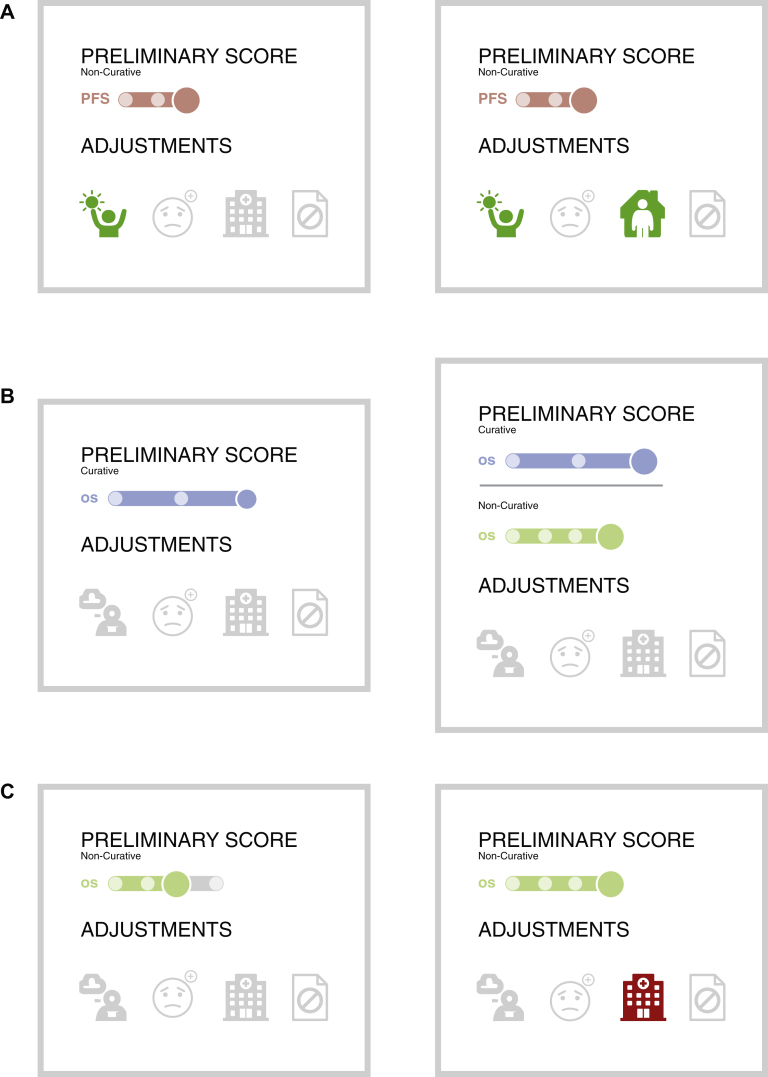
**Scenarios of visualisations presented during the prototype survey. (**A) Survey question: which therapy do you perceive as providing better clinical benefit? Most responders (75%) preferred the right visualisation. (B) Survey question: which therapy do you perceive as providing better clinical benefit? Most respondents (72%) preferred the left visualisation. (C) Survey question: which therapy do you perceive as providing better clinical benefit? Responders were divided into two choices (Preferences: 59% left and 41% right).

In comparing visualisations of treatment with equivalent ESMO-MCBS scores but different adjustment criteria (scenario 1), 75% (*n* = 41) of respondents perceived a treatment meeting two adjustment criteria as more beneficial than one meeting a single criterion (Figure 2A). This suggests that when a treatment qualifies for an adjustment in two ways, both should be visualised even if the ESMO-MCBS score is only upgraded once.

In scenario 2, we presented two visualisations for an immunotherapy treatment of metastatic melanoma that received a dual score of A/4: one depicted only the curative score, while the other showed both the curative and non-curative score. The visualisation with both scores was incorrectly perceived by 72% of respondents (*n* = 39) as indicating a higher clinical benefit, revealing a common misconception. This suggests the need for an alternative display method for dual-scored treatments in the future to avoid such misunderstandings, as having dual scores does not inherently imply greater benefit than treatments with a curative intent alone (Figure 2B). Scenario 3 included two studies with the same final ESMO-MCBS scores but with a different initial assessment: the first had a preliminary score with no adjustment, while the second had a higher preliminary score with a subsequent downgrade due to toxicity. Opinions varied, with 59% of respondents (*n* = 32) viewing the treatment without the toxicity downgrade as more beneficial (Figure 2C). This divergence aligns with our hypothesis that individuals' perceptions of clinical benefit vary based on the treatment profile presented.

#### General feedback (section 5)

Overall, the visualisations were interpreted accurately, with most respondents agreeing that they effectively convey a therapy's clinical benefit. However, nearly half of the respondents (48%) reported confusion, suggesting that additional background knowledge enhanced correct interpretation. They recommended that icons should be clarified at the outset, and a legend should be included to assist in comparing therapies, particularly when overall survival and progression-free survival are factored into the scores.

Importantly, feedback from the field-testing highlighted a unique perspective on the QoL adjustment icons, where rain could be interpreted as a positive outcome, and sunshine was seen negatively, contrary to their intended meanings. This feedback has prompted a redesign of these icons to eliminate any potential misinterpretation, as well as the inclusion of a legend.

### Final amendments to the design

In response to the survey insights, the visualisation team enacted comprehensive revisions (Figure 1, bottom panel). These included a standardised colour palette and highlighted the final score in both the detailed and summarised visualisation.

Legends now accompany each ESMO-MCBS visualisation, detailing icons and colour codes, and a glossary includes all abbreviations. The header has been updated to feature the treatment agent and trial name, when relevant. The information section has been expanded to include therapeutic indication, experimental, and control arm details, along with options to download the visualisation in PDF format.

Several refinements to the adjustment section and icons were also made.

1. Adjustment sliders have been enhanced with descriptive legends. For instance, an ‘Early crossover’ note now clarifies the rationale for the other adjustments slider’s position when such event occurs in a clinical trial.
2. The icon for negative QoL now depicts a cloud with thunder instead of rain.
3. The ‘Adverse events impacting daily well-being’ and ‘Adverse effects resulting in hospitalisation’ sliders have been consolidated into one, named ‘Serious and disabling adverse effects’.
4. The colour-coding of the slider knob and icon are now both coloured to enhance interpretability when the slider is positioned to the right or left.

The summarised version was also amended.

**Figure 3:**
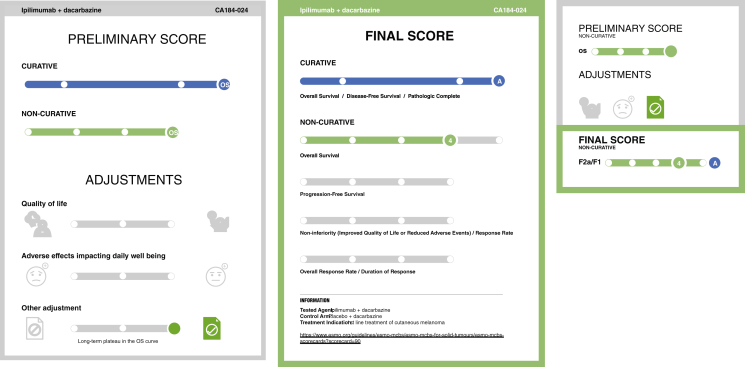
**Double score visualisation amendments.** This visualisation with the double scoring applies only in cases where two evaluation forms are used, specifically for form 2a treatments with remarkable survival benefit.

1. Previously, the icons were only coloured when they influenced the score; currently, when a treatment shows no improvement in quality of life, the QoL icon is displayed in black.
2. The upcoming ESMO-MCBS version (v2.0) will outline the ‘Serious and disabling adverse effects’ icon for curative treatments, regardless of its impact on the score.
3. Additionally, a single-line slider now visually represents dual scores based on user feedback (Figure 3).

### Evaluation survey

Following the above-mentioned updates, the visualisations were incorporated into the ESMO-MCBS Scorecards in June 2023. Accompanying them, a brief evaluation survey was launched to gather user interaction with the visualisations and their components, such as the legend and abbreviation glossary. The survey also sought feedback on any aspects of the visualisations that might be confusing or inappropriate when conveying a treatment’s clinical benefit.

Promoted via ESMO’s social media and at the ESMO Congress 2023 in Madrid, the survey collected 118 responses from 48 countries between June 2023 and January 2024. While most participants were medical oncologists (78%), patient advocates (4%), industry representatives (3%), and governmental health officials (2%) also contributed responses.

The feedback was overwhelmingly positive: 93% found the visualisations accessible, 92% easily located the legend, and 88% has no trouble finding the abbreviations. Between 80% and 90% of respondents did not find any part of the visualisations confusing. However, 18% of respondents indicated the preliminary score section was unclear due to the absence of actual numbers on the slider, and 16% had difficulties with the adjustment icons, although some noted the legend was helpful.

Respondents appreciated the ability to download the visualisations, with 79% intending to use them in presentations. No respondents reported the visualisations as offensive, and 86% rated them as moderately to extremely effective in communicating the clinical benefit of treatments. In conclusion, the feedback from the final evaluation survey shows that the ESMO-MCBS visualisations are generally considered a user-friendly and effective tool for visually communicating clinical benefits derived from clinical trial data.

## Discussion

Feedback indicated that those familiar with the ESMO-MCBS understood the visualisations well, while others less familiar found some elements unclear. Design improvements were made based on this feedback and the final visualisation was incorporated into the ESMO-MCBS Scorecards. A final survey showed that the majority of respondents found the tool to be accessible and no parts of the visualisation offensive. Similarly, the majority of respondents confirmed the tool to be effective at communicating the clinical benefit of a treatment and rarely found parts of the visualisation confusing, confirming the general understandability of the tool.

This is not the first visualisation tool to illustrate data about anticancer treatments. For instance, the National Comprehensive Cancer Network Evidence Blocks conveys a US expert panel’s recommendation for anticancer treatments by assessing five key factors: affordability, efficacy, safety, quality, and consistency.^20^ It features a block design with up to five blocks for each category, with more blocks indicating a better medicine's evaluation. Another example is the Drug Abacus, an interactive tool that evaluates cancer medicines, offering a comparative analysis of the manufacturer’s pricing for the US and UK markets, considering the treatment’s safety, innovation, cost, rarity of the disease, and overall disease burden.^21^ It allows users to modify variables to see how their preferences align with health care spending. The ESMO-MCBS visualisation is distinct from both of these, as it evaluates treatments derived from a clinical trial rather than evaluating the clinical trial itself. Furthermore, the score can be systematically derived from the clinical trial outcomes in contrast with a score derived from expert opinion.

ESMO-MCBS scores are already available on the ESMO website^18^ for >450 anticancer trials. It is hoped and anticipated that this added visual tool will further facilitate the accessibility and understandability of ESMO-MCBS by patients, doctors, and other stakeholders.

While spontaneous stakeholder feedback has been valuable during the development of the visualisations, implementing a more structured and continuous feedback mechanism would ensure their alignment with evolving user needs. Thus, we invite users to share their feedback as they engage with the forms and Scorecards by writing to mcbs@esmo.org.
