# Supplementary material for: The ESMO-Magnitude of Clinical Benefit Scale (ESMO-MCBS) visualisation: picturing the evidence of clinical benefit of clinical trial data
Source: ESMO Real World Data Digit Oncol. 2025 Aug 26;9:100171. doi: 10.1016/j.esmorw.2025.100171 (PMC12836693; doi:10.1016/j.esmorw.2025.100171)
Supplement: Supplementary Material 1 [file mmc1.pdf]

ESMO-MCBS Visualisation

<u>Prototype 1</u>

Carlos Urzua

Rudolf Fehrmann

Elisabeth de Vries

Watermarked icons depcited here are illustrative examples that will be later replaced by commissioned visual assets

ESMO-MCBS v1.0 and v1.1

OXFORD ACADEMIC

ANNALS OF ONCOLOGY

Issues More Content ▾ Publish ▾ Purchase Advertise ▾ About ▾

EDITOR'S CHOICE

A standardised, generic, validated approach to stratify the magnitude of clinical benefit that can be anticipated from anti-cancer therapies: the European Society for Medical Oncology Magnitude of Clinical Benefit Scale (ESMO-MCBS) FREE

N. I. Cherny ✉, R. Sullivan, U. Dafni, J. M. Kerst, A. Sobrero, C. Zielinski, E. G. E. de Vries, M. J. Piccart

Ann Oncol (2015) 26 (8): 1547-1573. **DOI:** https://doi.org/10.1093/annonc/mdv249
Published: 30 May 2015 **Article history** ▾

Views ▾ PDF Cite Share ▾ Tools ▾

EDITOR'S CHOICE

ESMO-Magnitude of Clinical Benefit Scale version 1.1 FREE

N. I. Cherny ✉, U. Dafni, J. Bogaerts, N. J. Latino, G. Pentheroudakis, J.-Y. Douillard, J. Tabernero, C. Zielinski, M. J. Piccart, E. G. E. de Vries

Annals of Oncology, Volume 28, Issue 10, 1 October 2017, Pages 2340–2366, https://doi.org/10.1093/annonc/mdx310
Published: 04 September 2017

Views ▾ PDF Cite Permissions Share ▾

Aims

- A visualisation that informs patients, physicians, institutions and industry about the characteristics of a treatment that influence its clinical benefit.
- A visualisation that can be used to see the context of each treatment and their comparability to other potential alternative treatments.

Factors taken into account for ESMO-MCBS

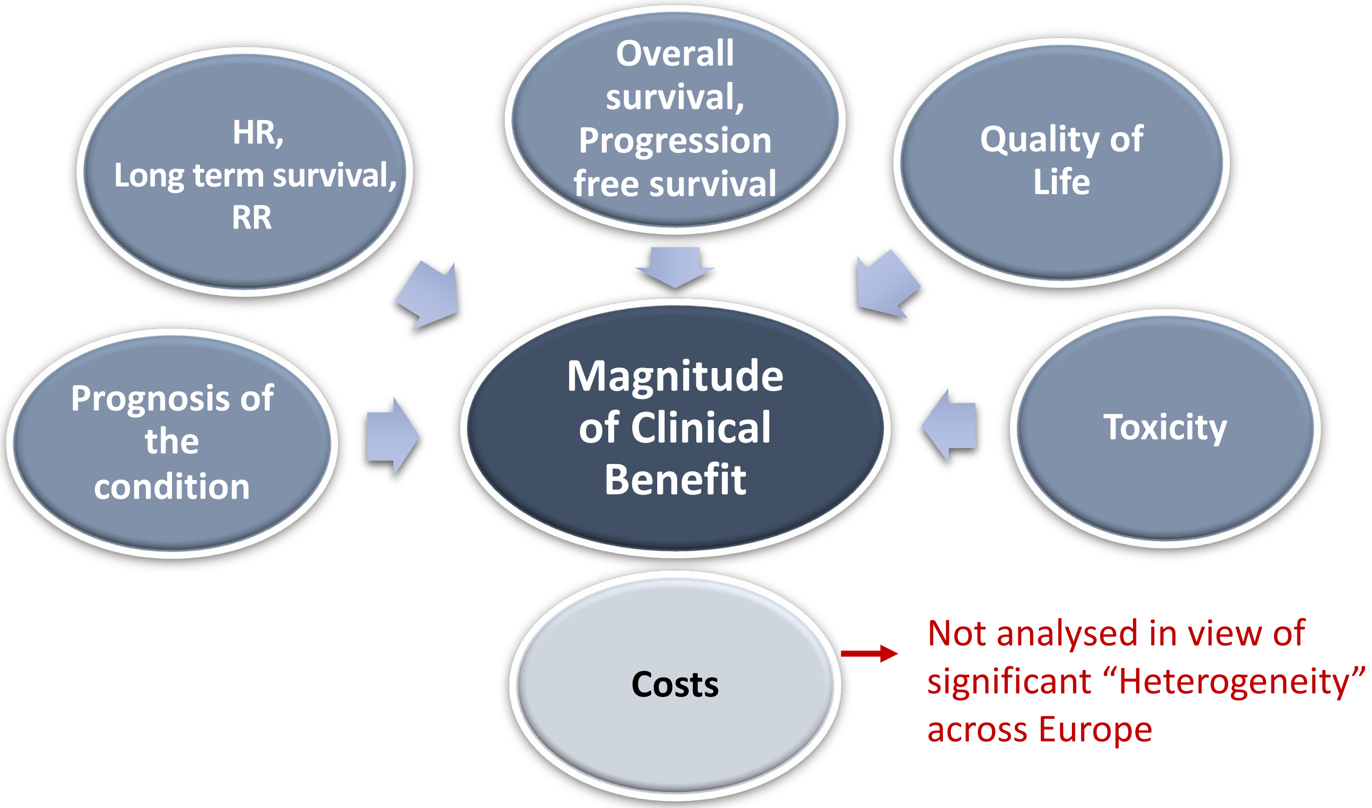

Underlying Premises ESMO-MCBS

1. Cure takes precedence over deferral of death
2. Direct endpoints such as survival and QoL take precedence over surrogates such as PFS or RR
3. DFS in curative disease is a more valid surrogate than PFS or RR in non-curative disease
4. Interpretation of the evidence for benefit derived from surrogate outcomes (such as PFS) may be influenced by secondary outcome data

Definition ESMO-MCBS substantial improvements

- Curative setting A & B or non-curative setting 5 & 4

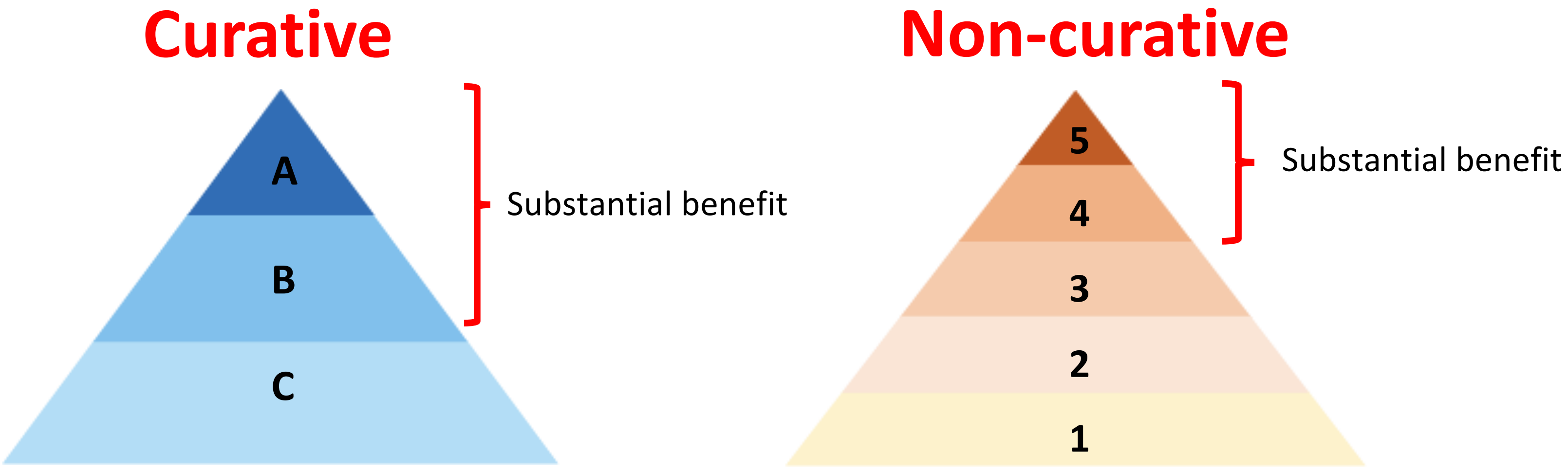

Methods

- The visualisation has two sections:
  - The right panel describes the final score.
  - The left panel describes the specific aspects that influenced that score.

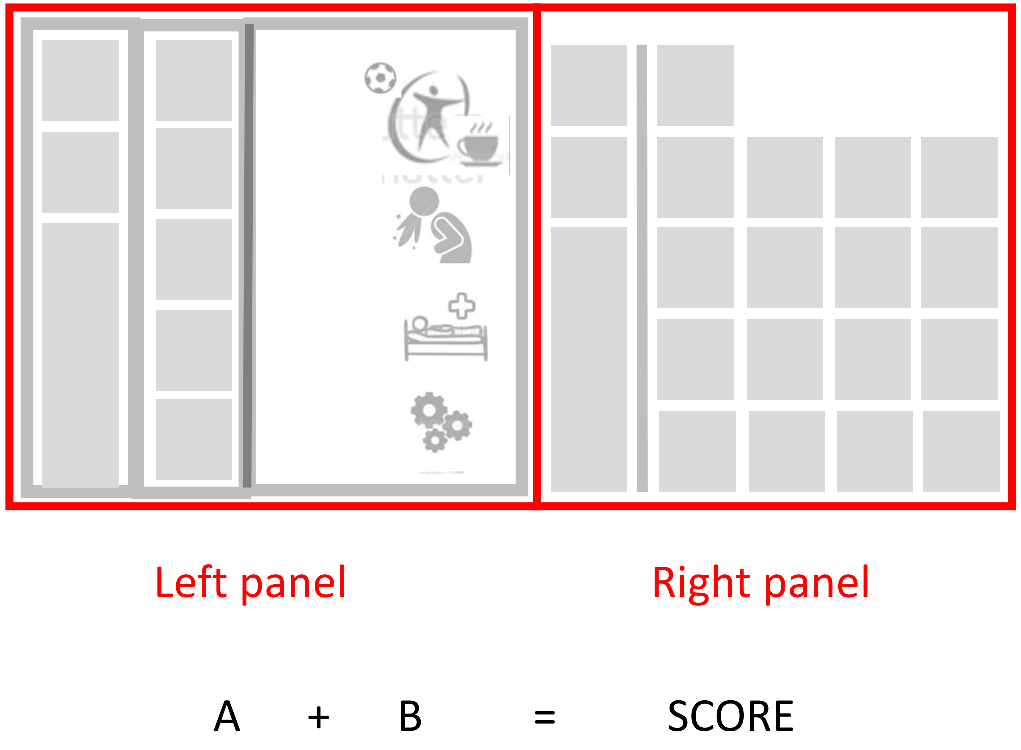

Methods

- The right panel is composed of columns:
  - Each column represents the different evaluation forms.

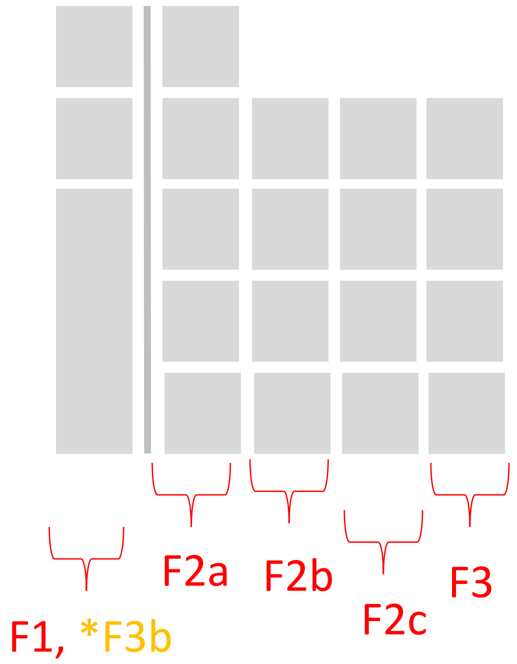

Methods

- The right panel:
  - Each form scores drugs using clinical trials that measured survival or other endpoints.

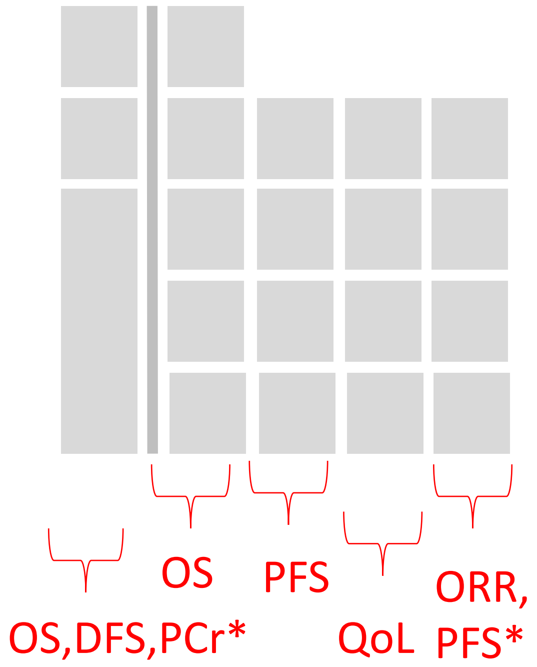

*Single arm studies

Methods

- The right panel:
  - The left vertical separator divides drugs with non-curative intent from drugs administered with curative intent.

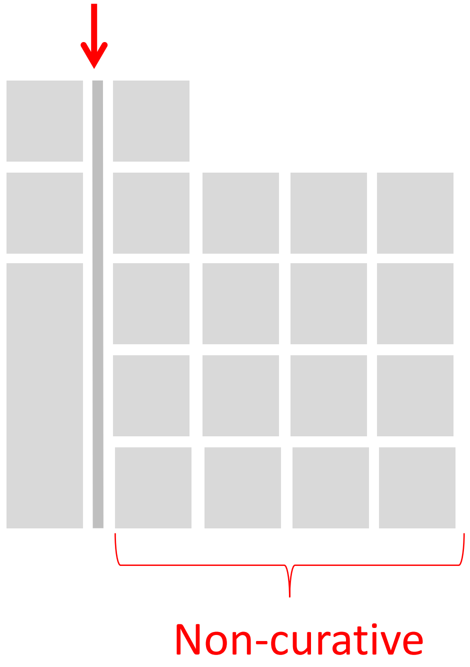

Methods

- The right panel:
  - When evaluating a drug, the column corresponding to its related form is the location where we illustrate the final score.
  - (e.g Form 2b)

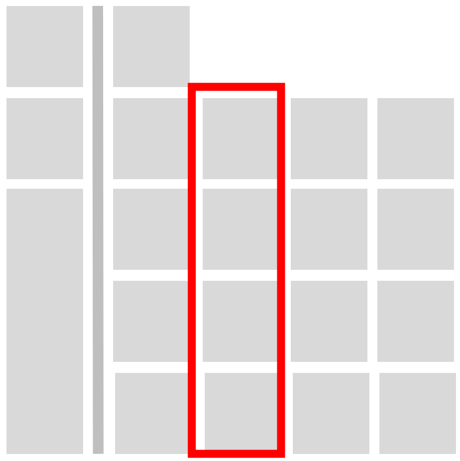

Methods

- The right panel:
  - To illustrate the score we use a vertical stack of blocks.
    - The height of the stack indicates how good the score is.
    - We use the color of the form to color the blocks.
    - We indicate the letter or number of the score obtained in the highest block of the stack.

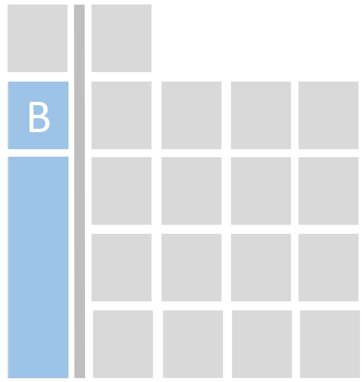

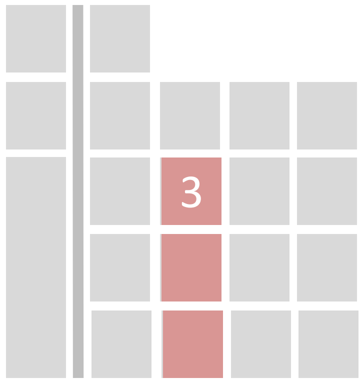

Methods

- The right panel:
  - If pending data from ongoing studies will modify the score we indicate it with a “?” in the potential form or score.

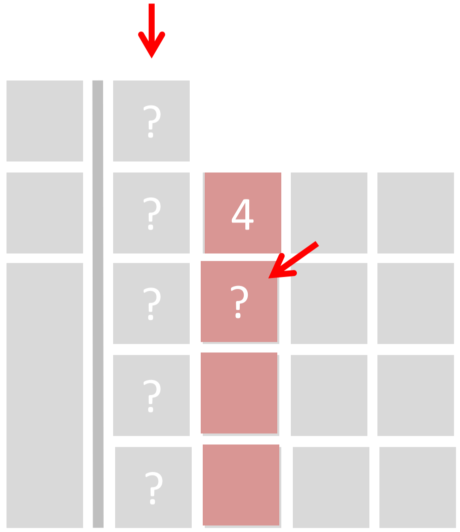

Methods

- The left panel has two sections:
  - A first section for the preliminary score.(left of the dark separator)
  - A second section for modifiers of the preliminary score (right of the separator)

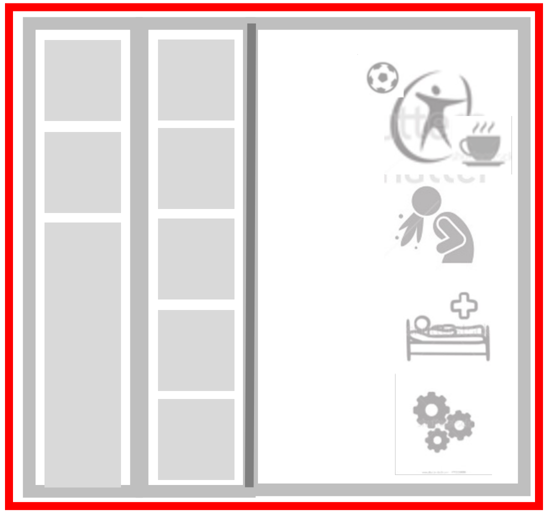

Left panel

Methods

- The left panel (First section):
  - Similarly to the right panel, we indicate with colored blocks the preliminary score of the drug.
  - The column location depends on the curative or non-curative intent of the drug

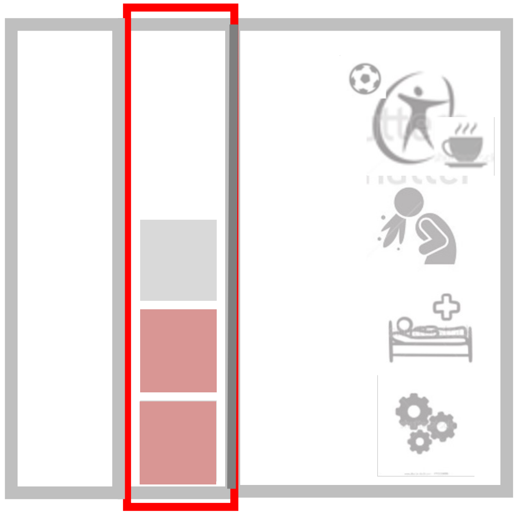

Preliminary score

Methods

- The left panel (First section):
  - We indicate the endpoint that was used to calculate the preliminary score on the highest colored block block.

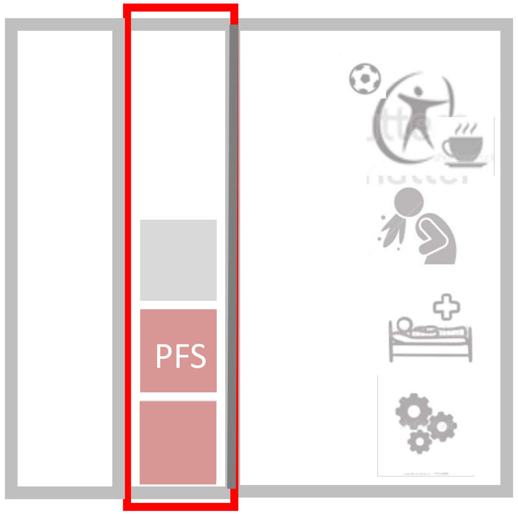

Preliminary score

Methods

- The left panel (First section):
  - We indicate the upgrading modifiers of the preliminary score that apply to that drug. (Smiley ☺)

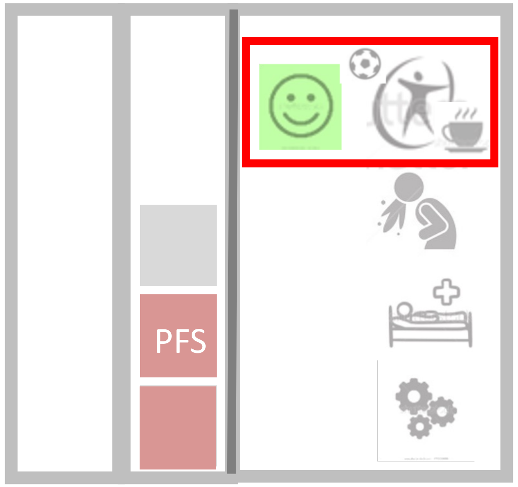

Methods

- The left panel (First section):
  - We indicate the downgrading modifiers of the preliminary score that apply to that drug. (Smiley ☹)

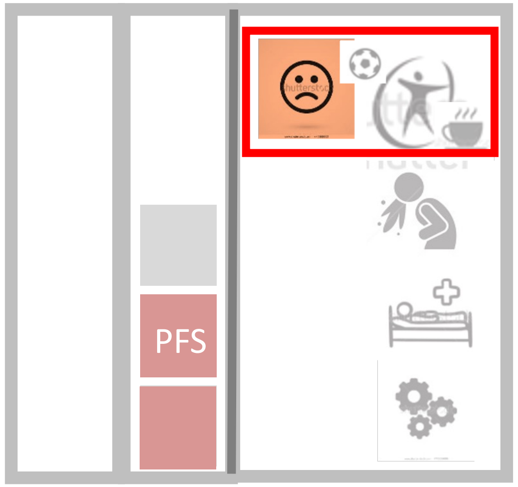

Results

- The ESMO-MCBS visualisation prototype 1
  - Example: An imaginary drug improves on Progression Free Survival and quality of life. Final score of 3 (Non-curative intent)

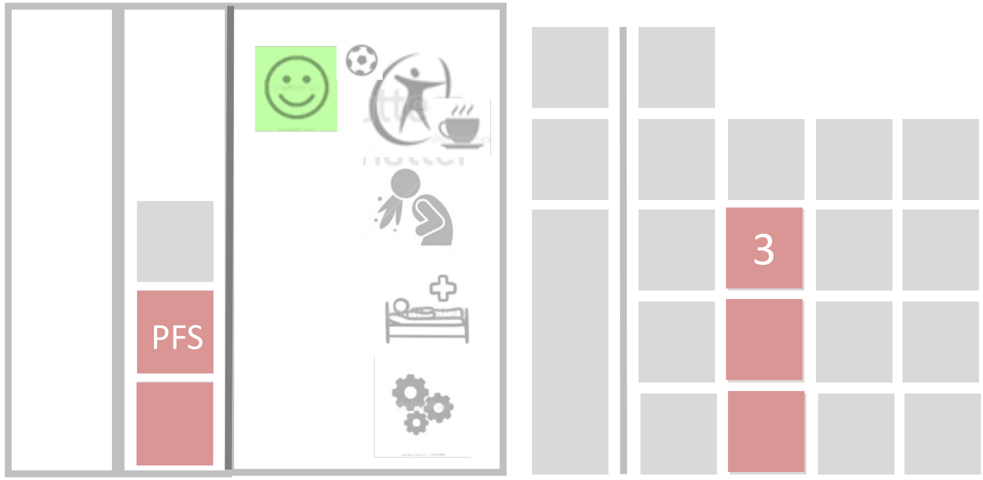

Imaginary Trial

Examples: Checkmate 141

- Reached a score of 4 *
    - *(All patients group)
- We can see that it is a therapy without curative intent that is a substantial improvement.
    - *It improves OS and QoL.*
    - *It may be scored as a curative therapy in the future.*

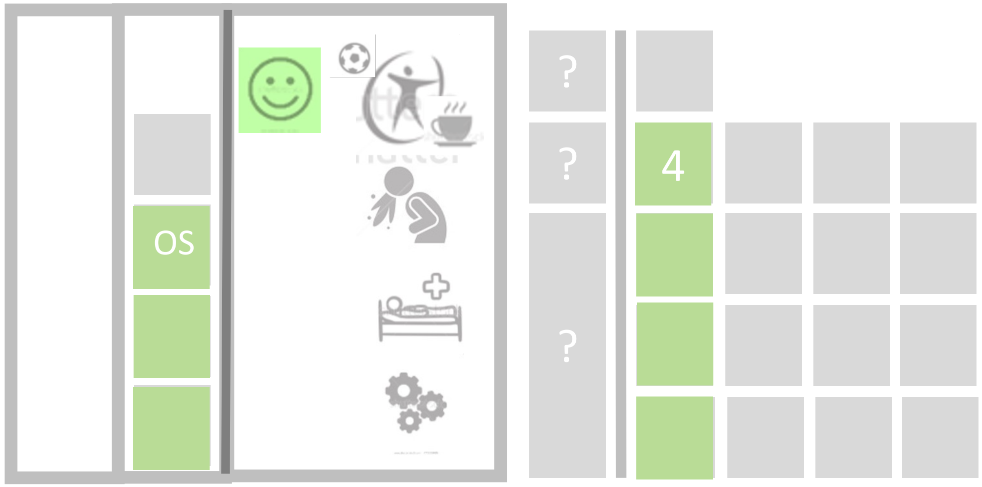

Checkmate 141

Examples: PALOMA-3

- Reached a score of 4
- We can see that it is a therapy without curative intent that is a substantial improvement.
    - *It improves PFS and QoL.*
    - *It has OS data pending, therefore its score or form may change in the future.*

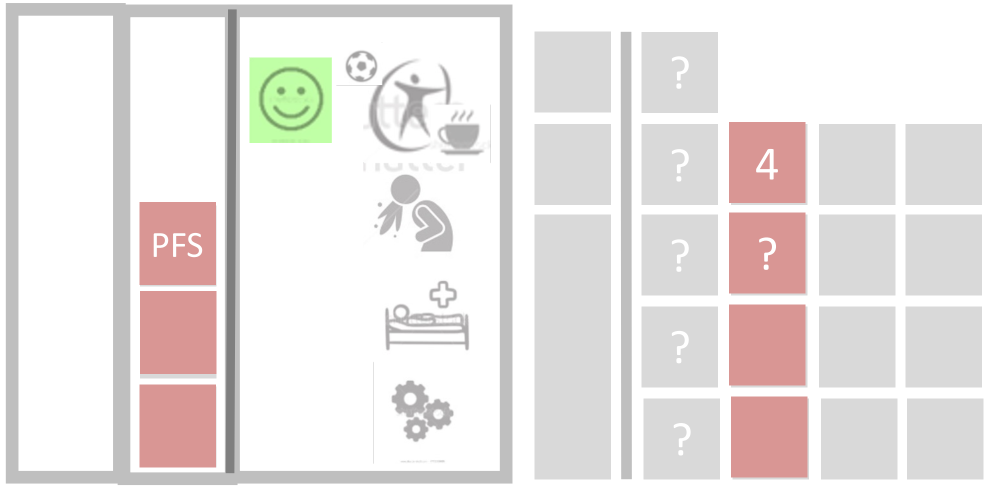

PALOMA-3

Discussion

- Both of the previous examples would have been difficult to distinguish without a visualisation.
  - *Both reached a score of 4*
  - *We could only assume that both drugs were substantial improvements without curative intent.*

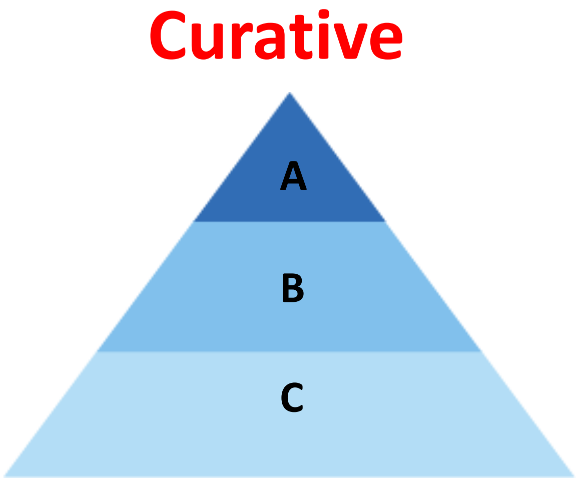

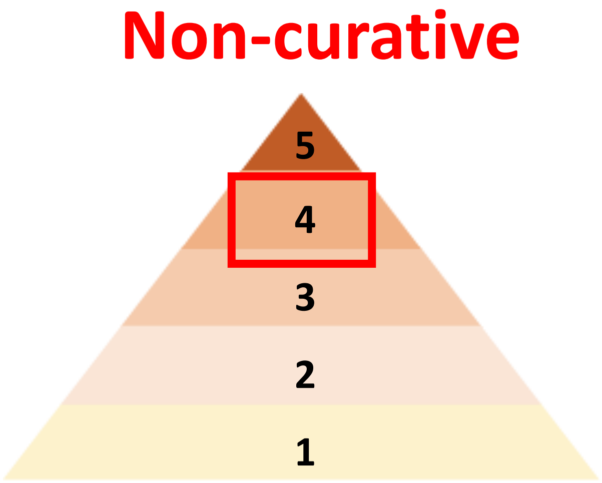

Aestethics and Visual Design of Visualisation

Visualisation Prototype

- Aims for Design:
  - To enable the comparison of treatment **benefits and disadvantages**.
  - Being informative <u>without causing alarm</u>.
  - Modular: Can be automatically constructed for future treatments with no loss in aesthetics.

- Target audience:
  - Patients, Physicians and non-expert stakeholders.

Visualisation Prototype

- Two main Objectives:

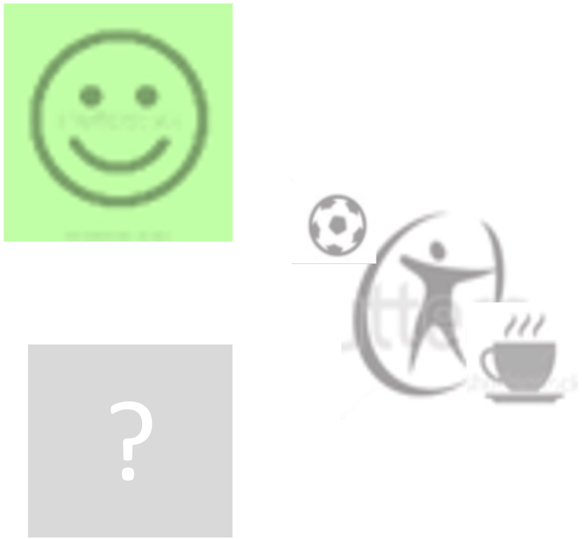

1. Designing appropiate icons. (3 prototypes per icon from which one will be revised 3 times)

2. Constructing the layout of the visualisation. (3 prototypes from which one will be revised 3 times)

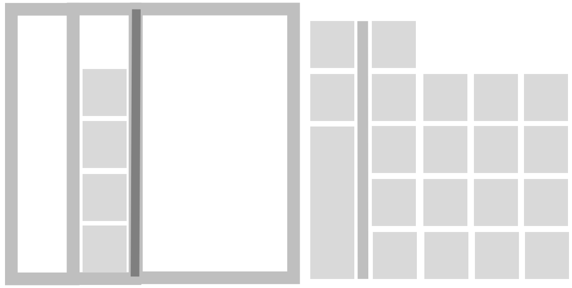

Constraints for the visualisation layout

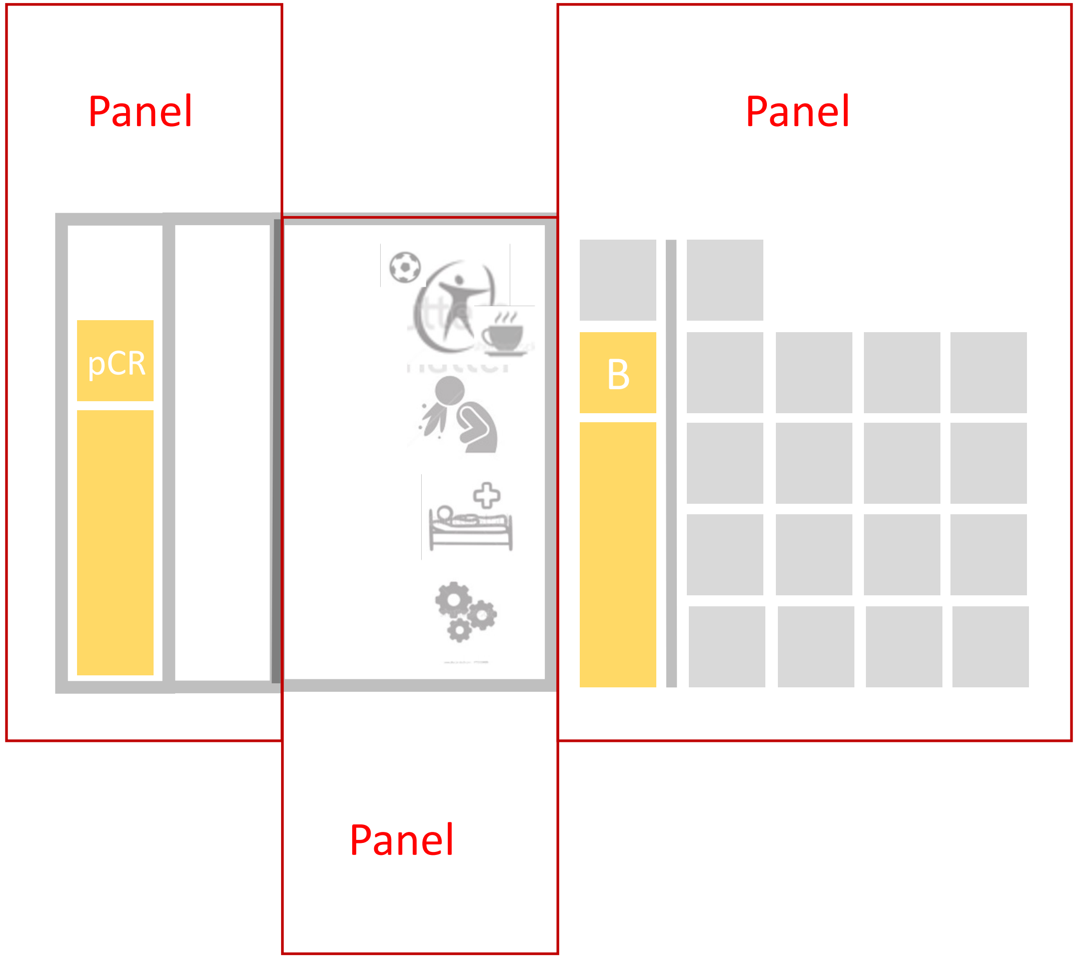

- Colors must be matching the color of the official forms.
- An increase in score from 1->2 should have the same visual impact as a change in a higher level (e.g. 4 -> 5)
- Panel relative positions can be changed.
- Icon placement can be changed within its subsection.
- Other subsection internal ordering remains fixed.
- Components **should be composable.** (For automatic generation)

Properties of the visualisation

- Attention should be prioritised:
  - In any setting:
    - The modifiers > the survival score > the final score

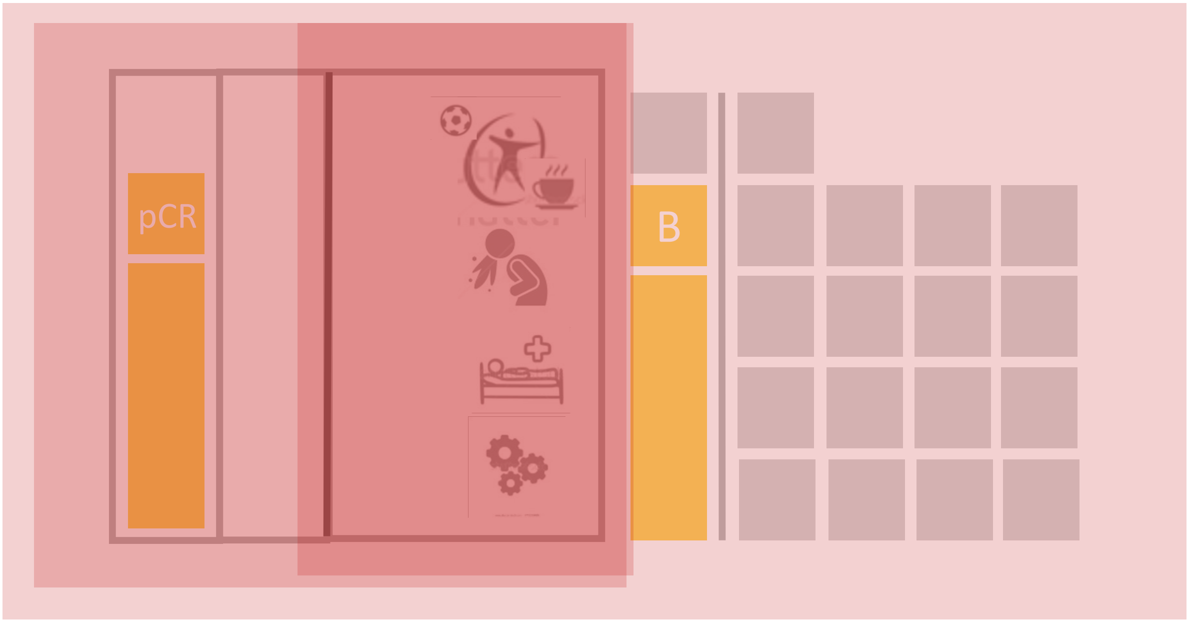

Properties of the visualisation

- Attention should be prioritised:
  - In the curative setting: Quality of life and long term toxicities should be prioritised.
  - In non-curative settings: Acute toxicities should be prioritised

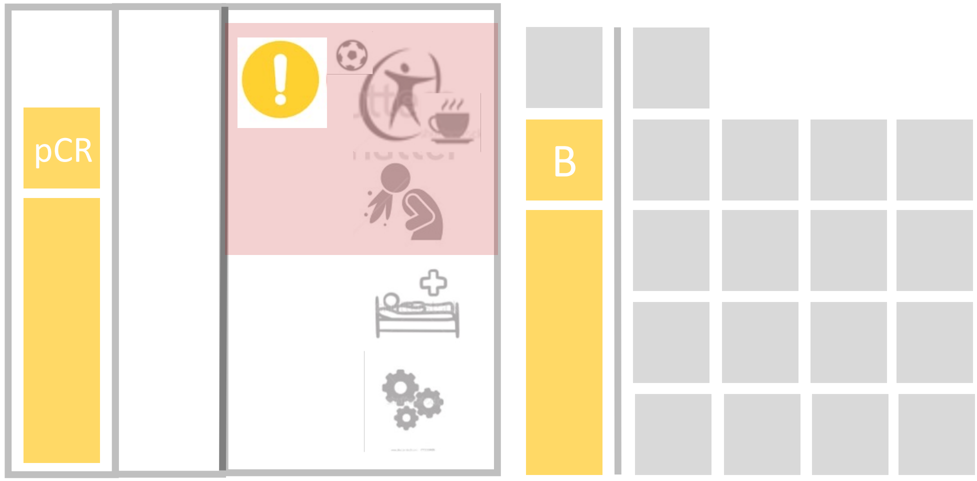

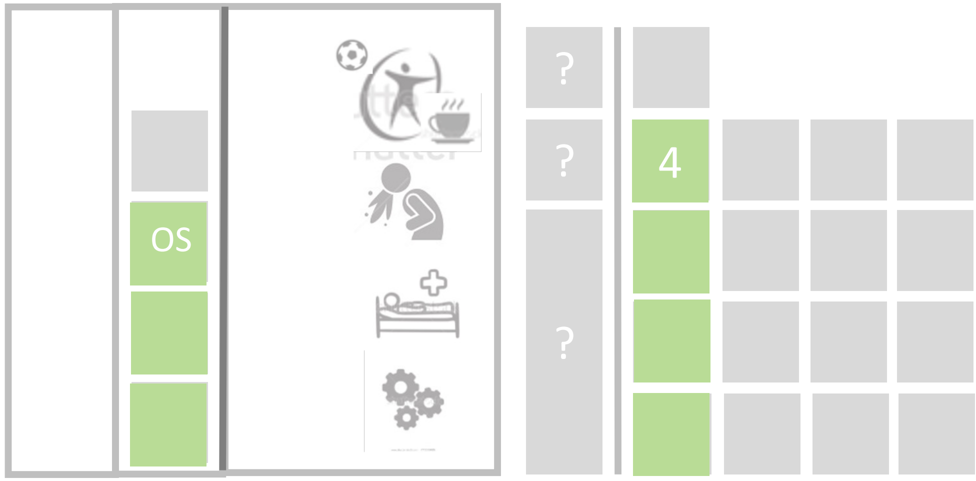

Imaginary trial

*Chronic neuropathy 15%

Visual guide for icons (e.g.)

- Icons:
  - **Quality of life.**
  - **Acute toxicities** that often warrant hospitalisation.
  - **Long term toxicities** that impact daily well being.
  - **Evidence superiority** of clinical trial. (Non-crossover).
  - **Interrogation sign** (Or other visual hint): Information will become available in the future.
  - **Smileys**: To indicate a positive , negative or neutral modifier in respect to the patient.

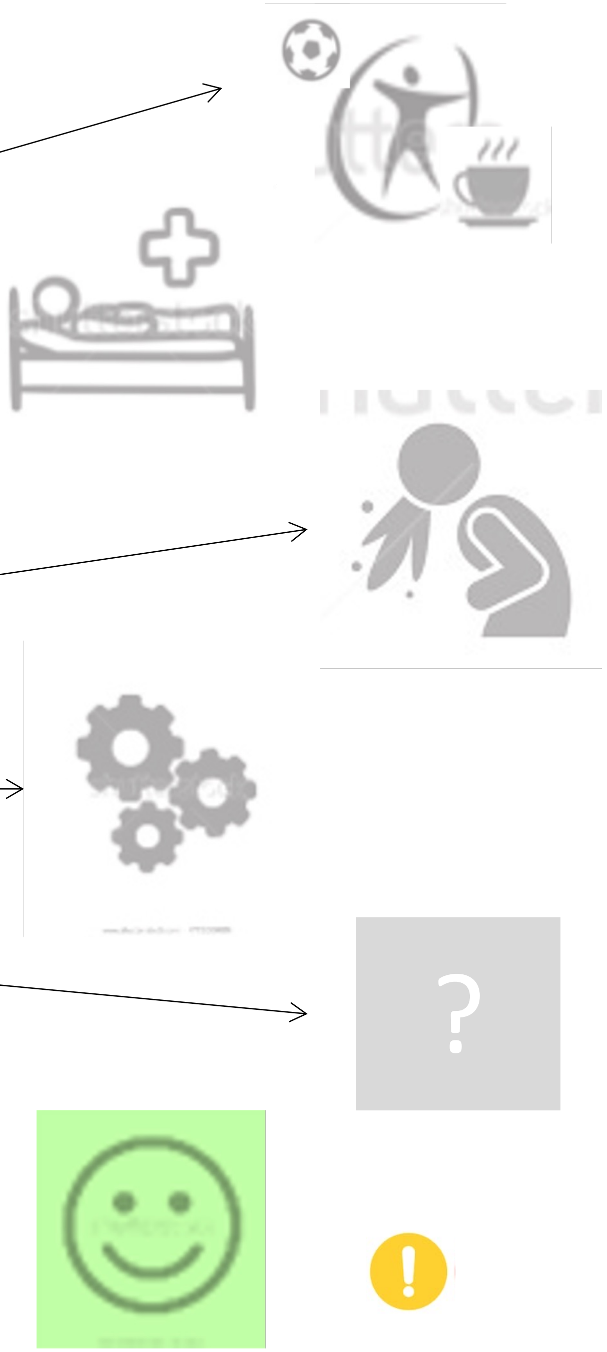

Thank You!

umcg

ESMO GOOD SCIENCE BETTER MEDICINE BEST PRACTICE

European Society for Medical Oncology
