# Supplementary material for: The ESMO-Magnitude of Clinical Benefit Scale (ESMO-MCBS) visualisation: picturing the evidence of clinical benefit of clinical trial data
Source: ESMO Real World Data Digit Oncol. 2025 Aug 26;9:100171. doi: 10.1016/j.esmorw.2025.100171 (PMC12836693; doi:10.1016/j.esmorw.2025.100171)
Supplement: Supplementary Material 2 [file mmc2.pdf]

Survey questions for ESMO-MCBS

Goal

- Swipe-left, swipe-right questions (11).
  - Testing basic understanding of the visualisation Testing icon interpretation
  - Testing potential pitfalls of the visualisation
  - Other feedback

Baseline understanding of visualisation

- Was the manual effective in communicating the visualisation meaning.

General layout questions

- Which section corresponds to the measured endpoint of benefit.
- Which section corresponds to the combined benefits or adverse effects of the treatment.
- Understandability of basic design messages.

Which section (left or right) encodes preliminary score?

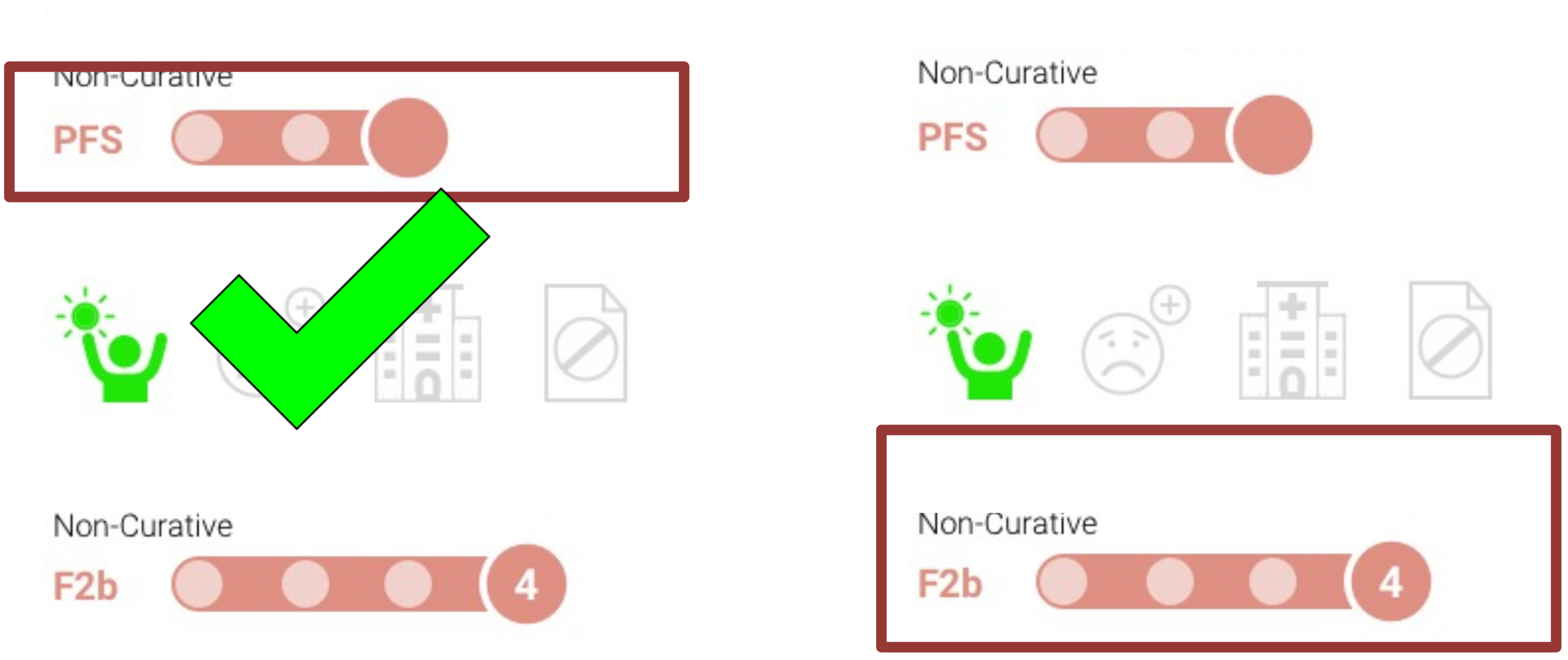

Which section (left or right) encodes adjustments to the score?

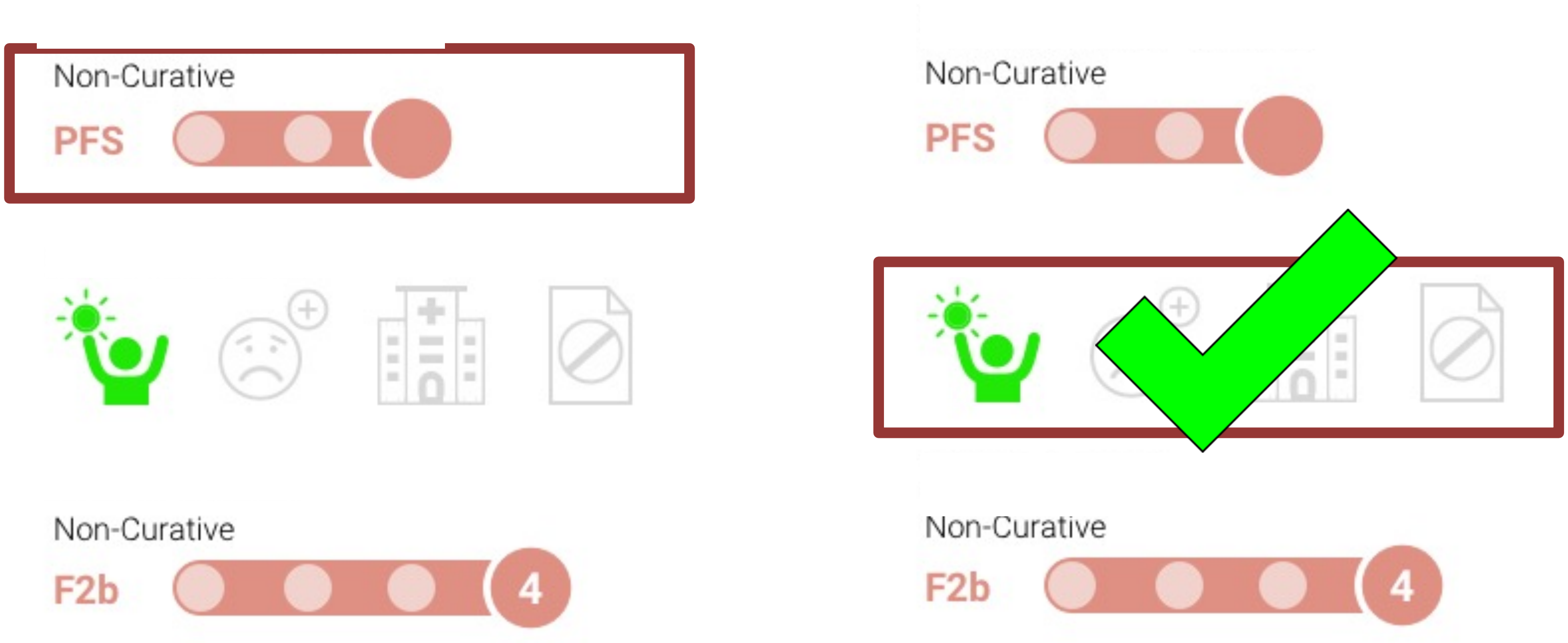

Which section (left or right) encodes the final score?

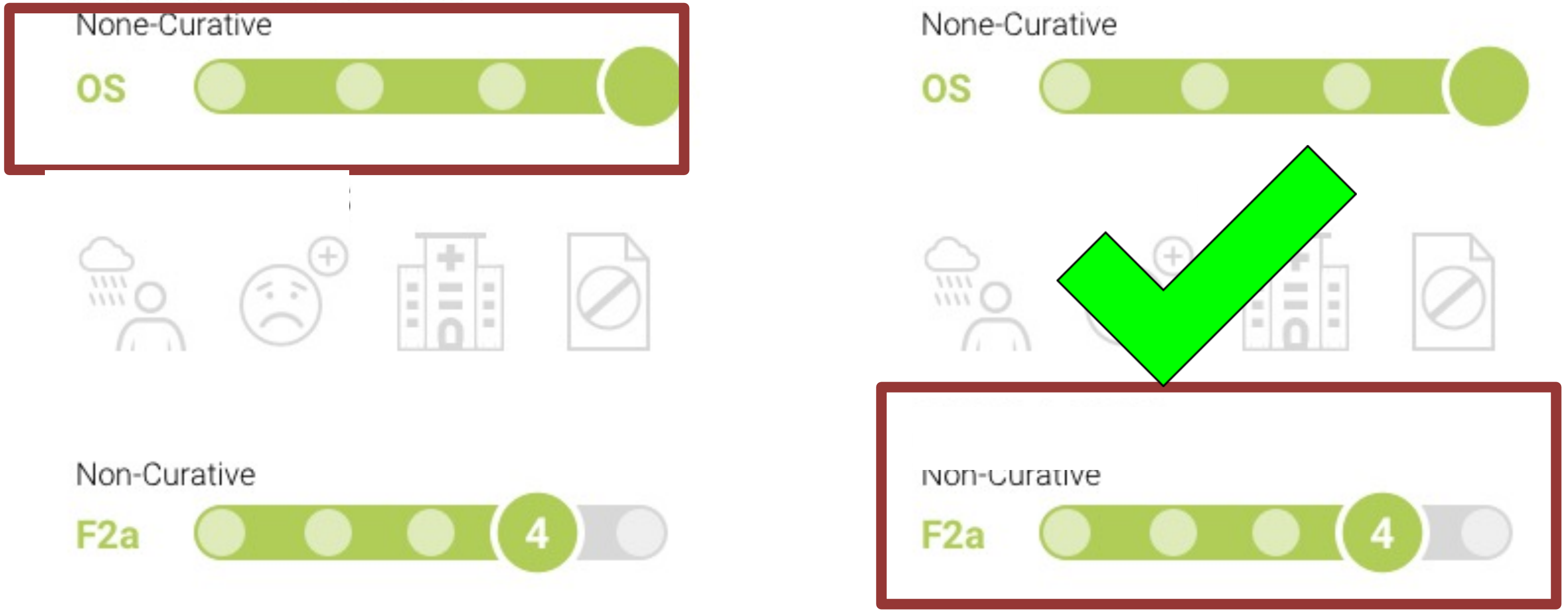

Which therapy (right or left) you percieve to have curative intent?

- Better with positional?

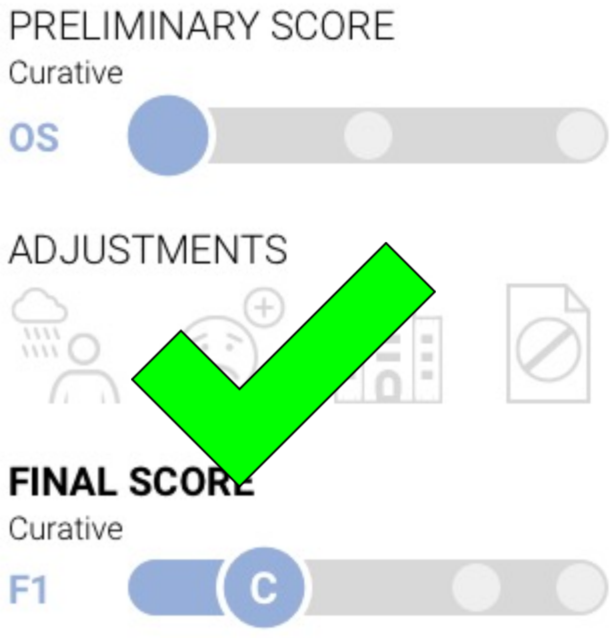

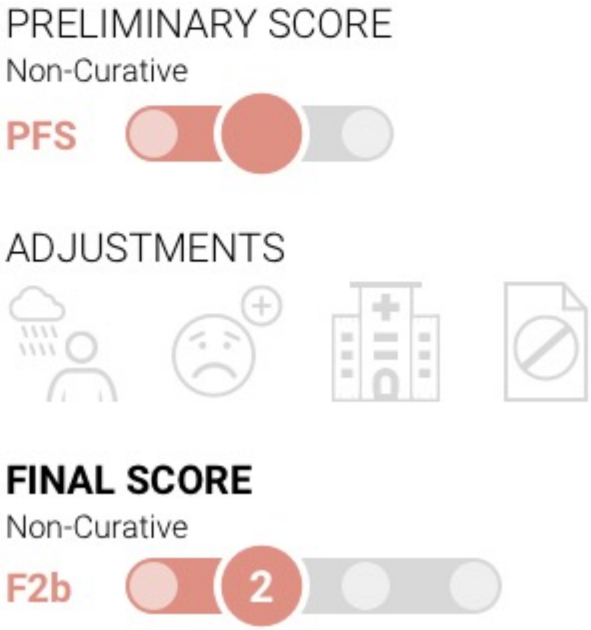

Which therapy (right or left) represents a substantial clinical benefit?

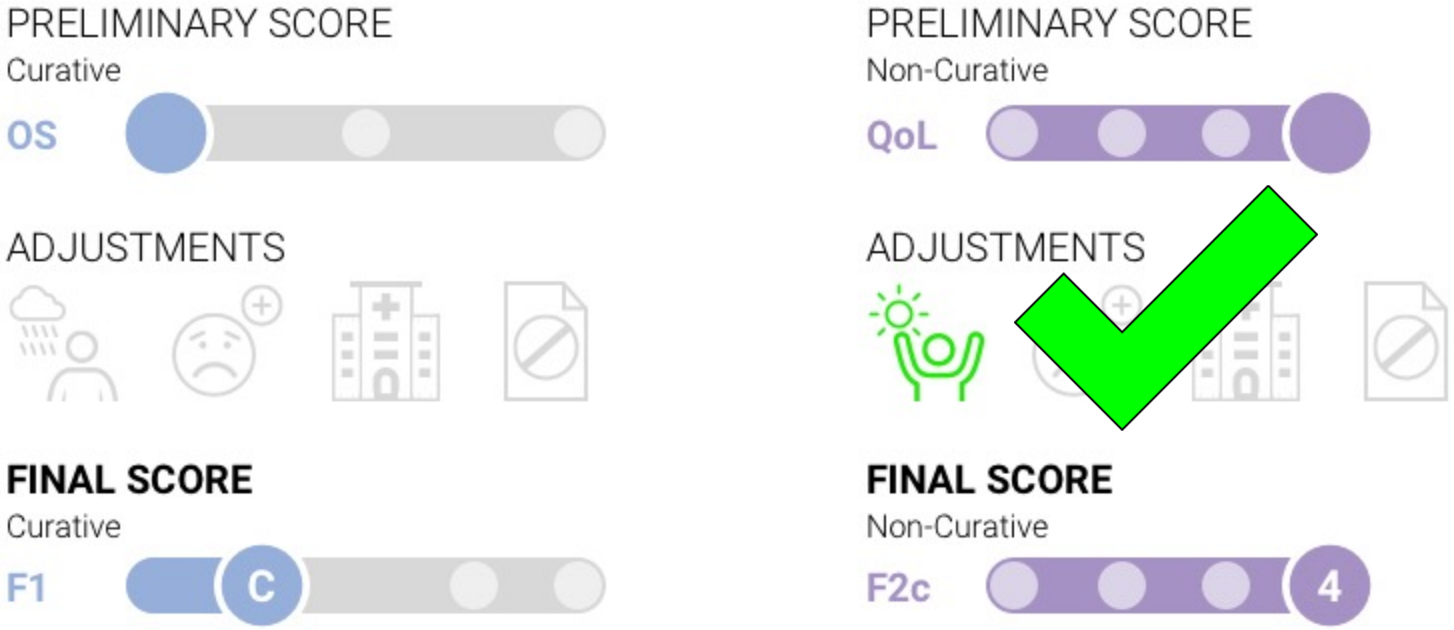

Which therapy (right or left) you percieve has pending data

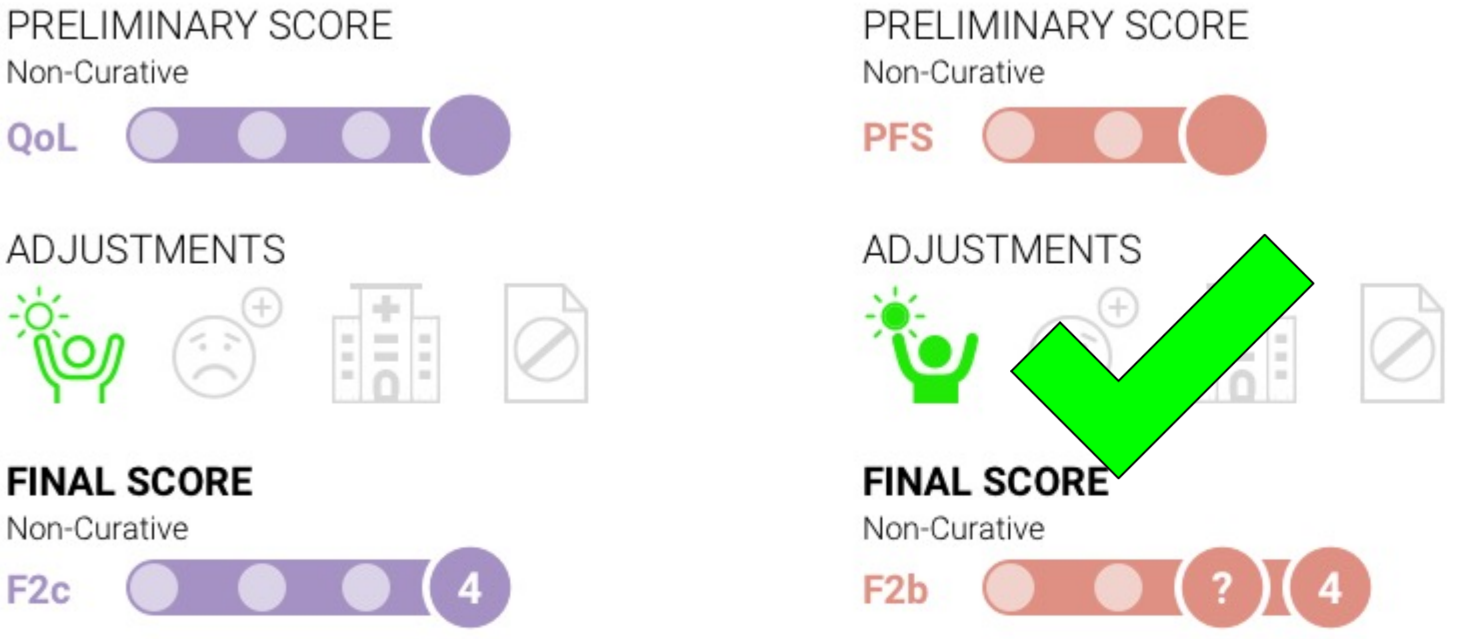

Which therapy (right or left) you percieve has better survival?

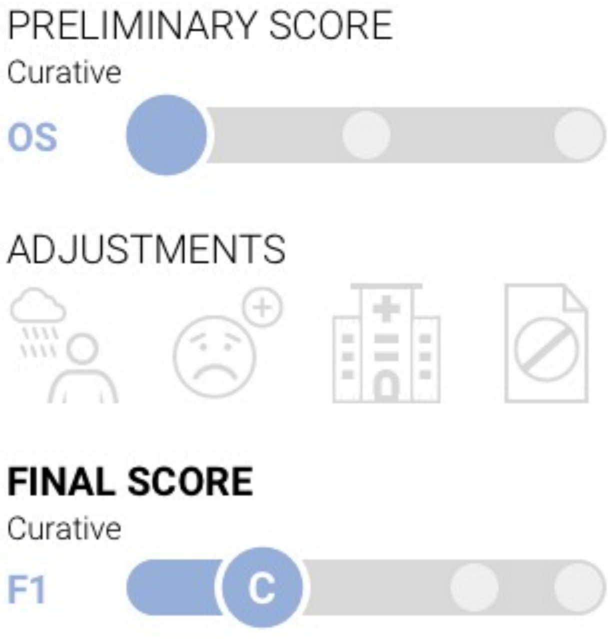

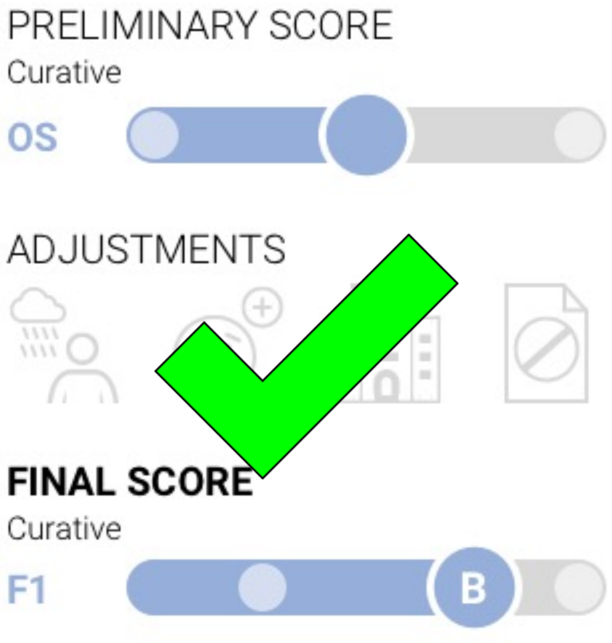

ICON interpretation

- Are icons being interpreted correctly

Which icon (up or down) you percieve communicates an improvement in quality of life?

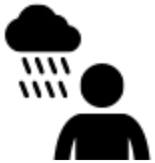

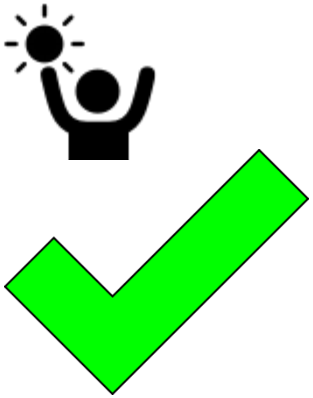

Which icon (up or down) you percieve communicates less ocurrence of side effects that result in hospitalization?

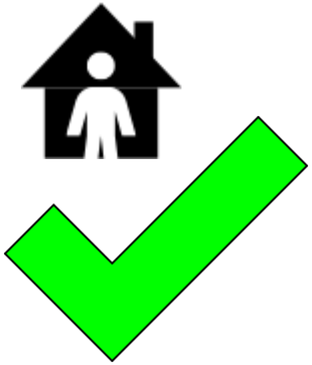

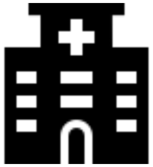

Which icon (up or down) you percieve communicates less ocurrence of side effects that impact daily well being?

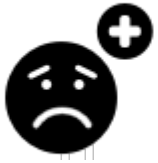

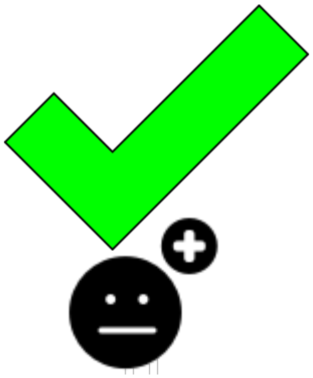

Which icon (up or down) you percieve communicates the achievement of a quality milestone?

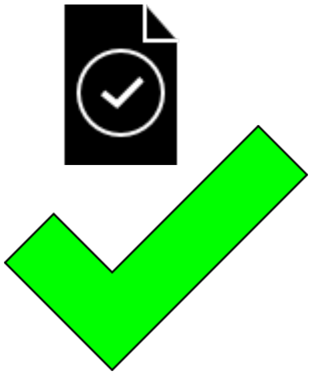

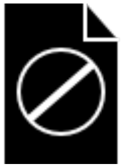

Which therapy (right or left) you percieve has modifiers that downgrade the preliminary score?

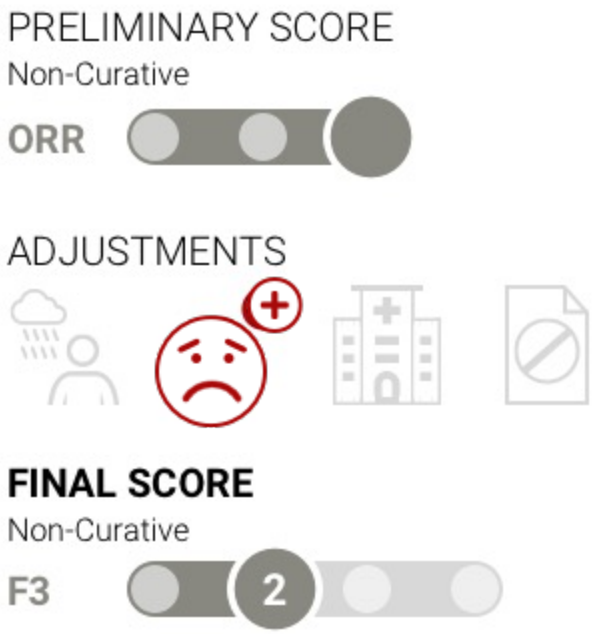

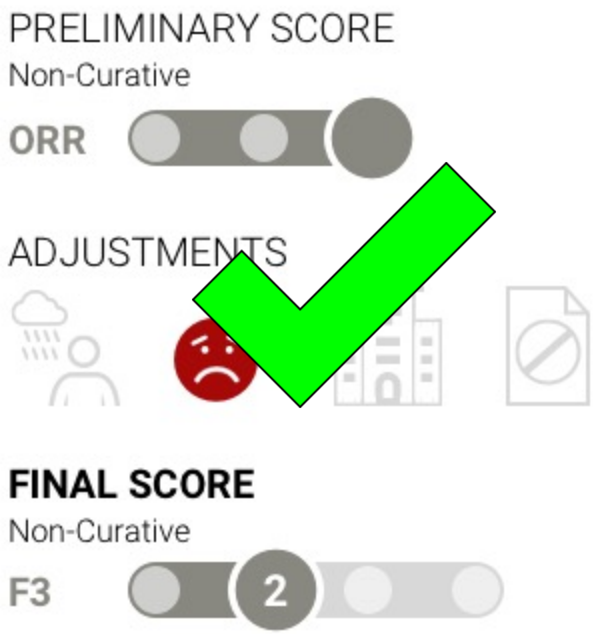

Which therapy (right or left) you percieve has modifiers that upgrade the preliminary score?

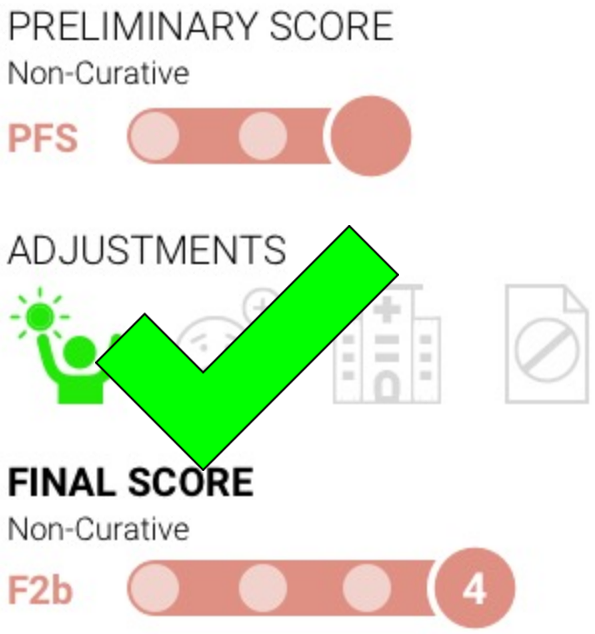

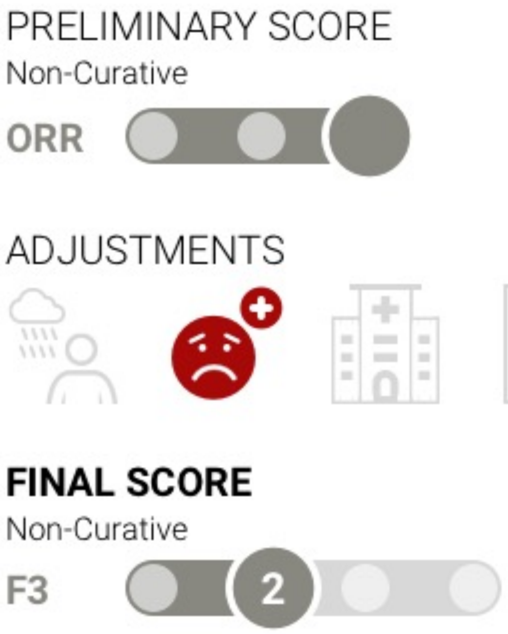

Which therapy (right or left) you percieve has reduced adverse efects that impact daily well being?

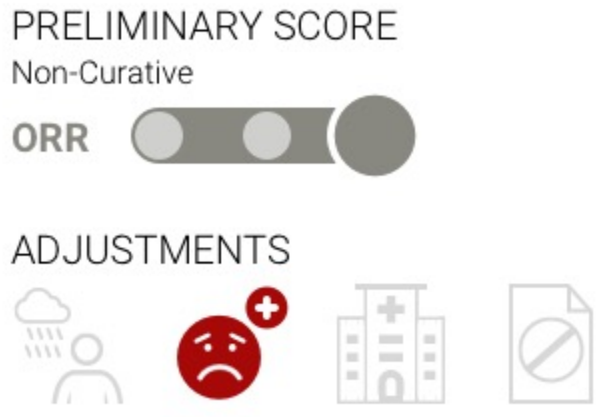

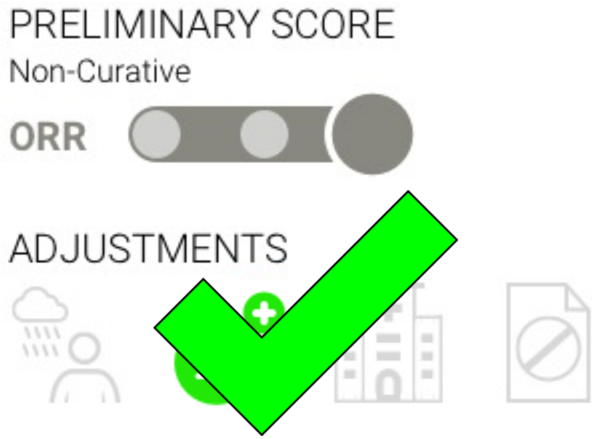

Opinion questions

- Potential misinterpretations of the visualisation.
- All have same final score. Do you prefer either one:

Which therapy (right or left) you percieve provides better clinical benefit?

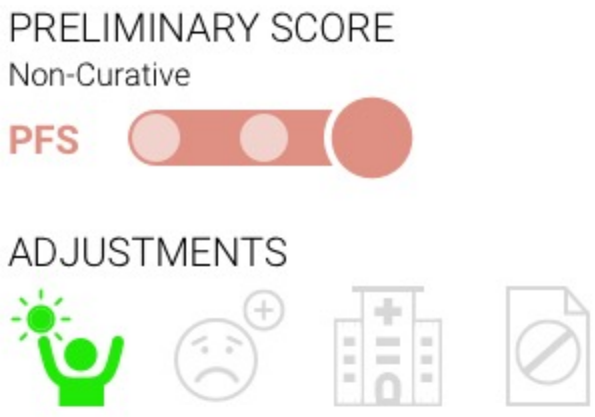

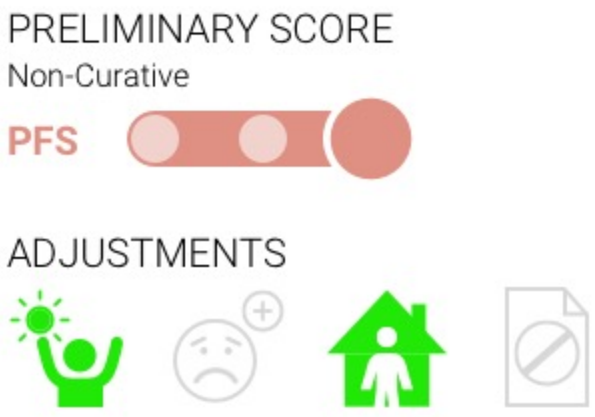

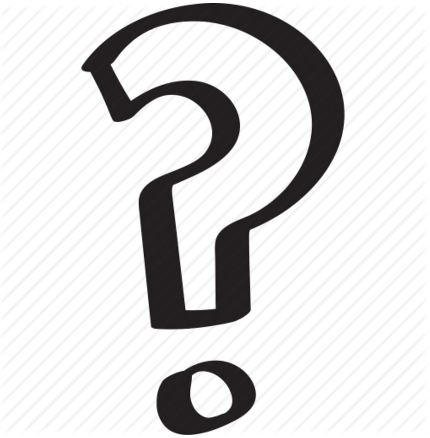

Which therapy (right or left) you percieve provides better clinical benefit?

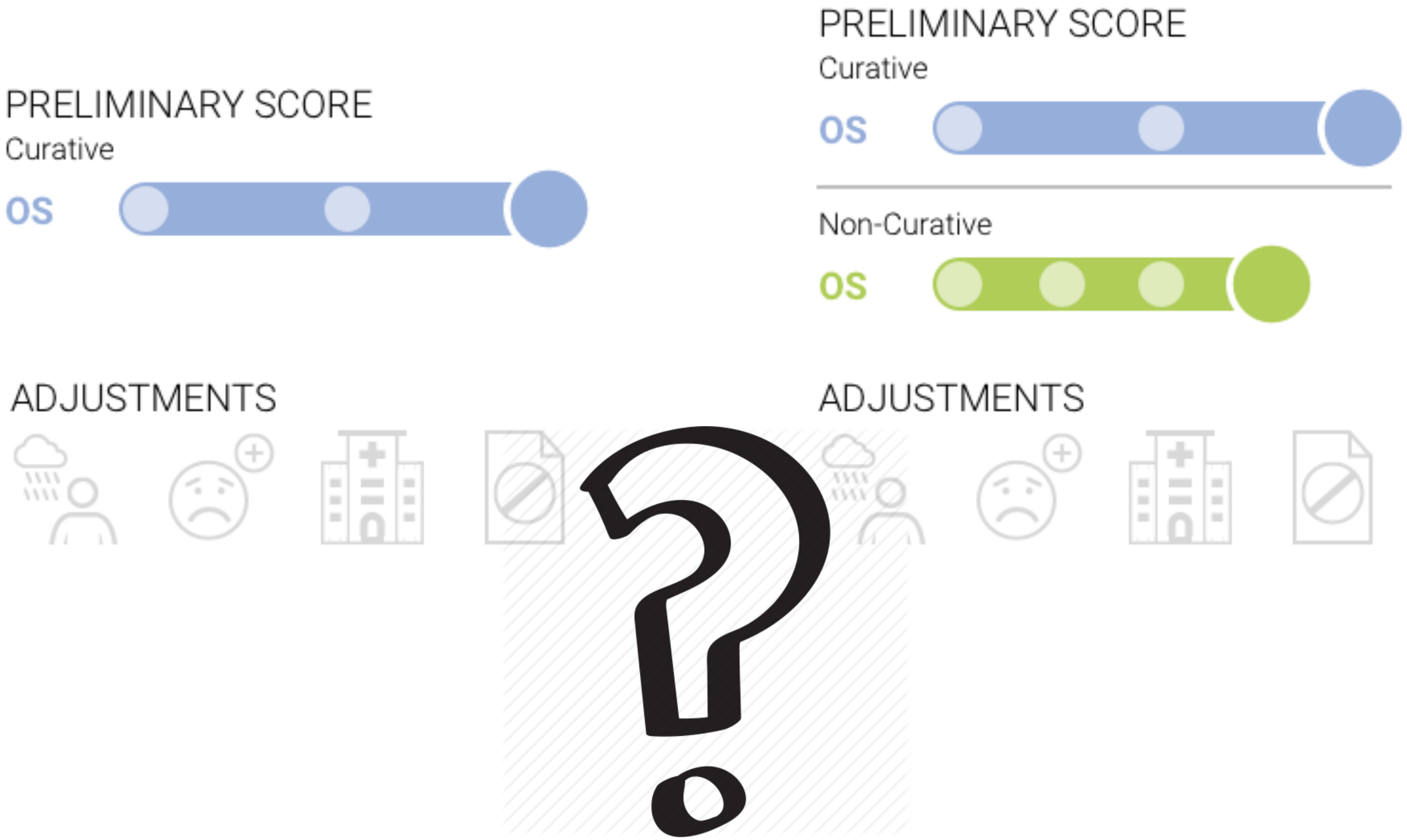

Which therapy (right or left) you percieve provides better clinical benefit?

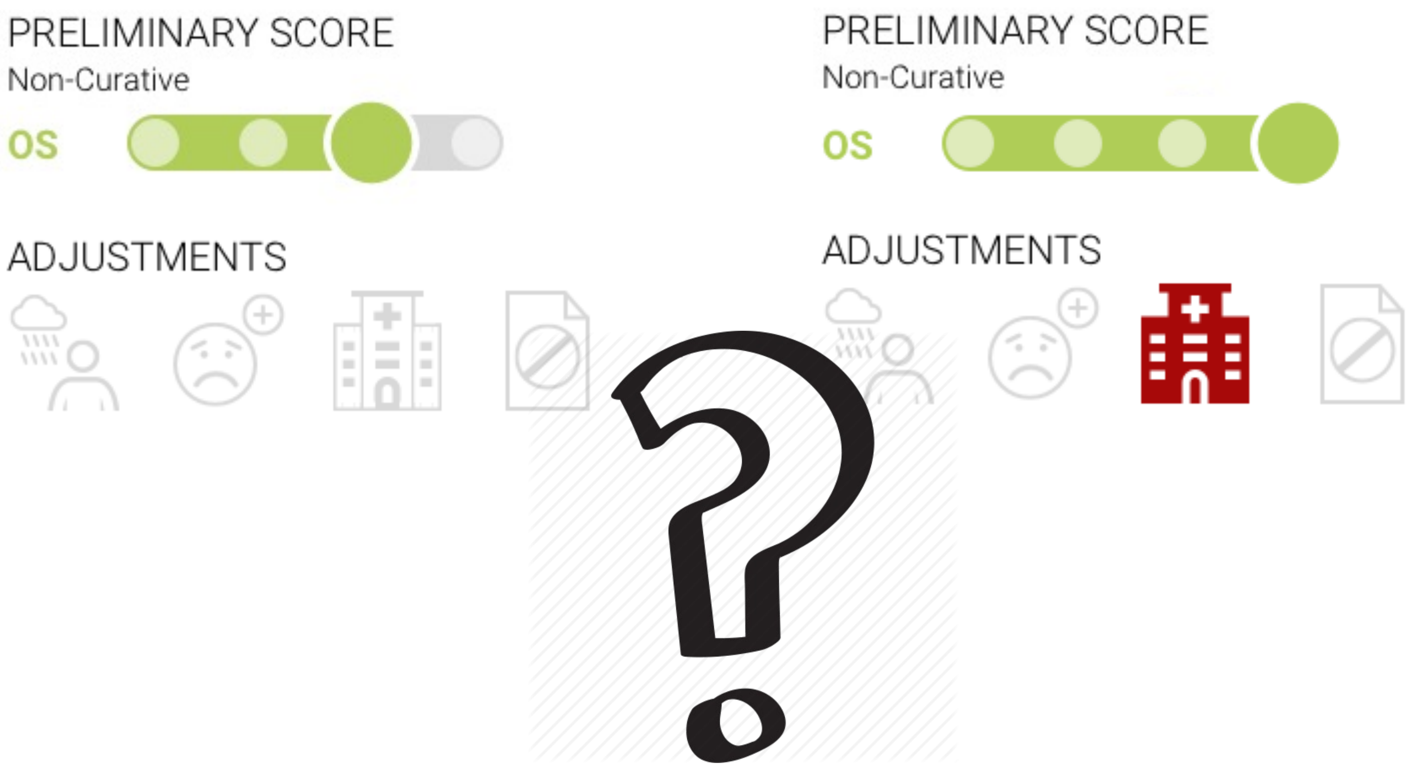

Which therapy (right or left) you percieve provides better clinical benefit?

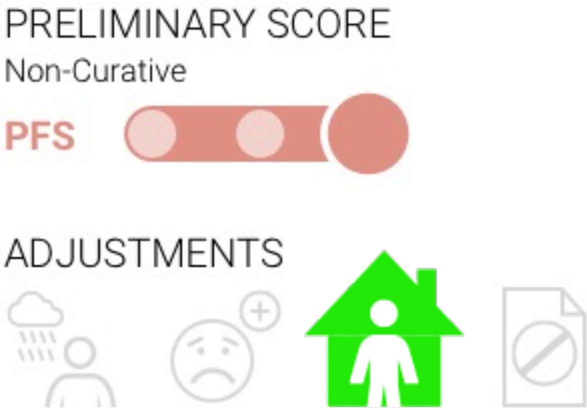

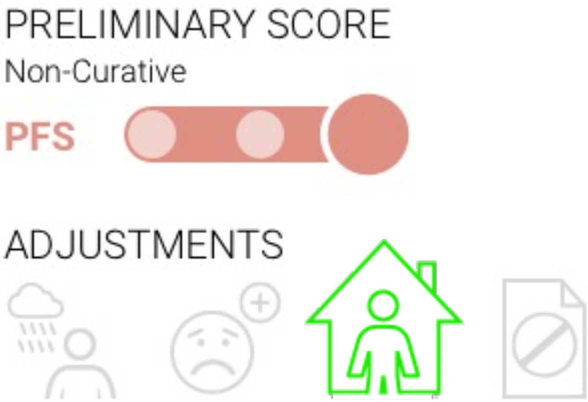

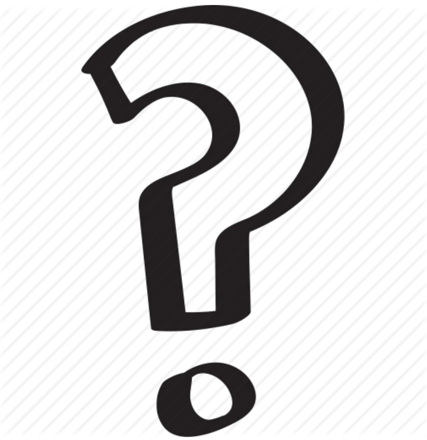

Further feedback

- Did you find parts of the visualisation confusing?
- Did you find parts of the visualisation offensive to patients?
