# Supplementary material for: The ESMO-Magnitude of Clinical Benefit Scale (ESMO-MCBS) visualisation: picturing the evidence of clinical benefit of clinical trial data
Source: ESMO Real World Data Digit Oncol. 2025 Aug 26;9:100171. doi: 10.1016/j.esmorw.2025.100171 (PMC12836693; doi:10.1016/j.esmorw.2025.100171)
Supplement: Supplementary Material 3 [file mmc3.pdf]

ESMO-MCBS Visualisation

Tutorial

ESMO

GOOD SCIENCE
BETTER MEDICINE
BEST PRACTICE

European Society for Medical Oncology

- The visualisation has three sections:
    1. The preliminary score panel describes the evaluated outcome.
    2. The adjustments panel describes the specific aspects that may influence the score.
    3. The final score panel.

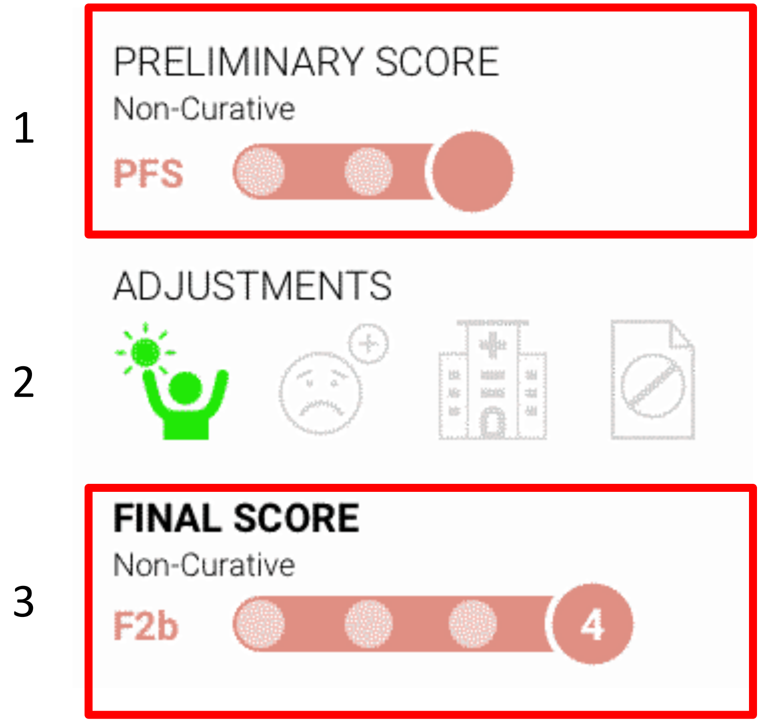

Detailed Version

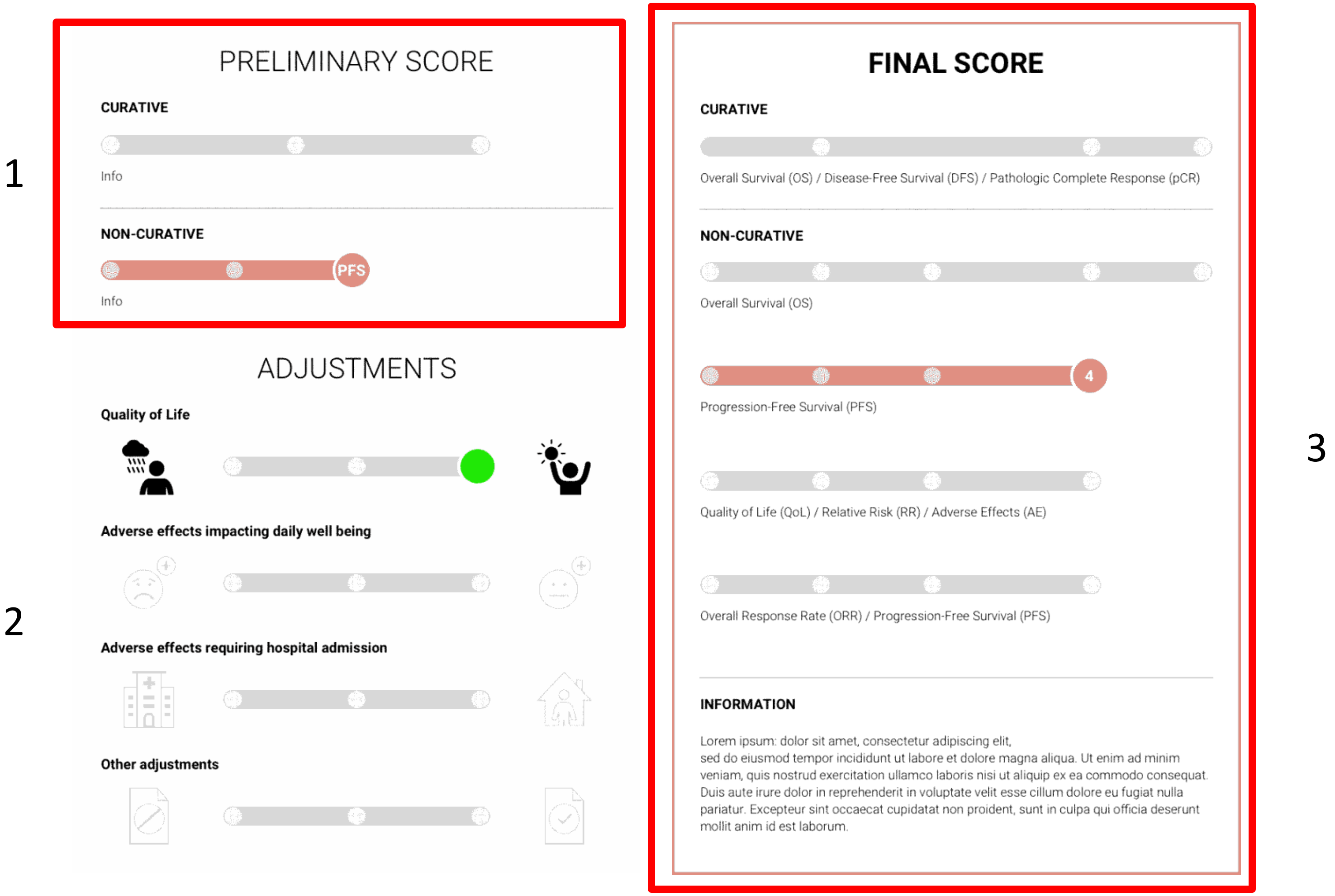

Detailed Version

- The final score panel is composed of sliders:
  - Each slider represents different evaluation forms.
  - Each form scores clinical trials with different endpoints.

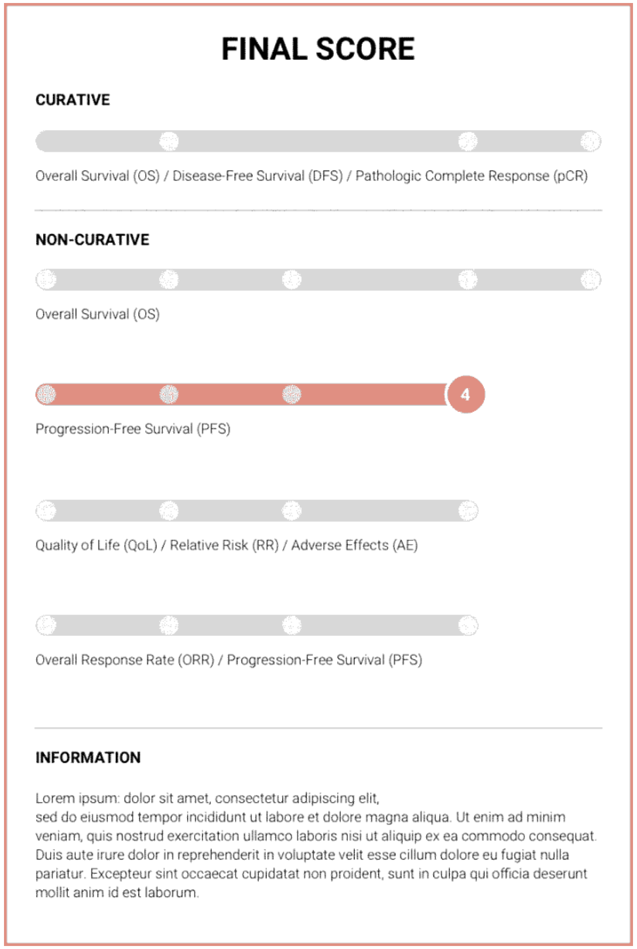

Detailed Version

- The final score panel:
  - The better the score is, the more the slider is filled.
  - The horizontal separator is in between forms that score trials with curative and non-curative intent.

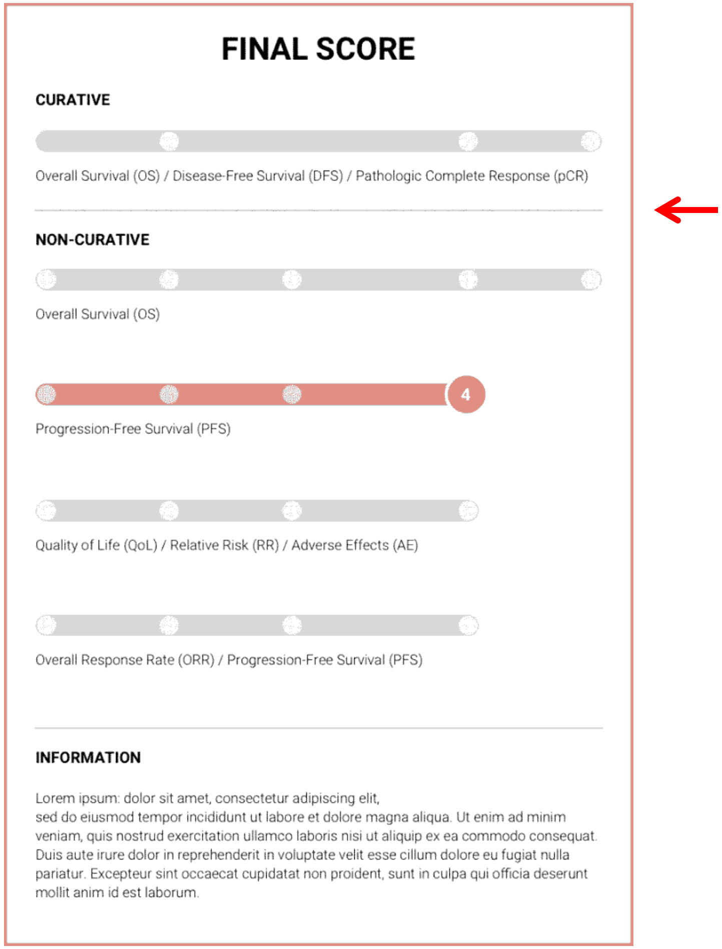

Detailed Version

- The final score panel:
  - The topmost scores (A,B,5,4) are considered substantial clinical benefit.

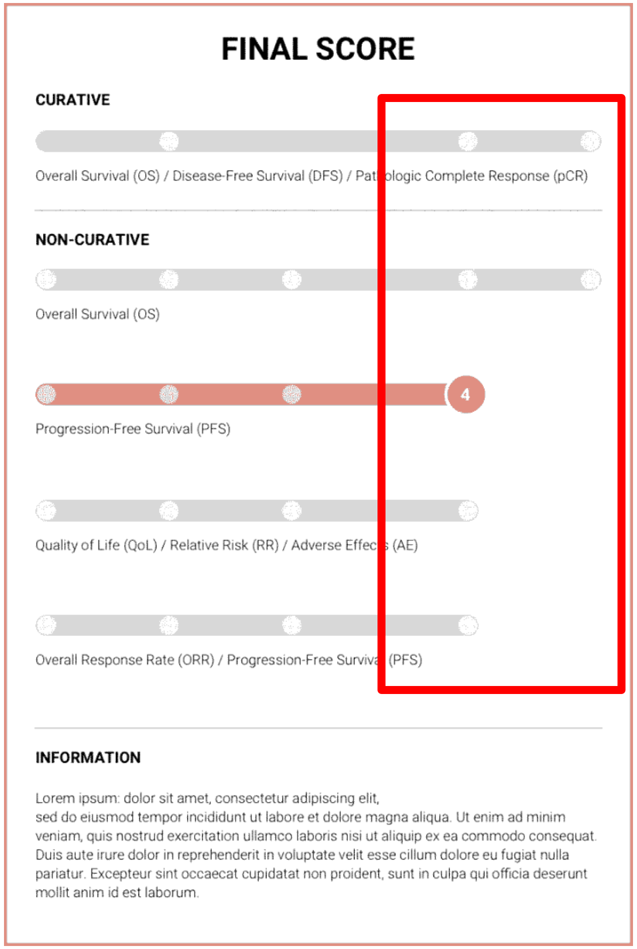

Detailed Version

- The final score panel:
  - If pending data from ongoing studies will upgrade or downgrade it is indicated it with a “?” at the potential future score.

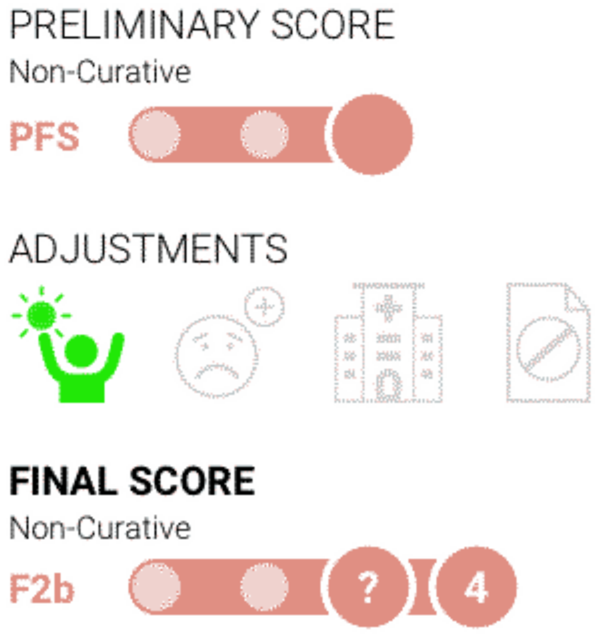

- The preliminary score panel:
  - The evaluated outcome is depicted.

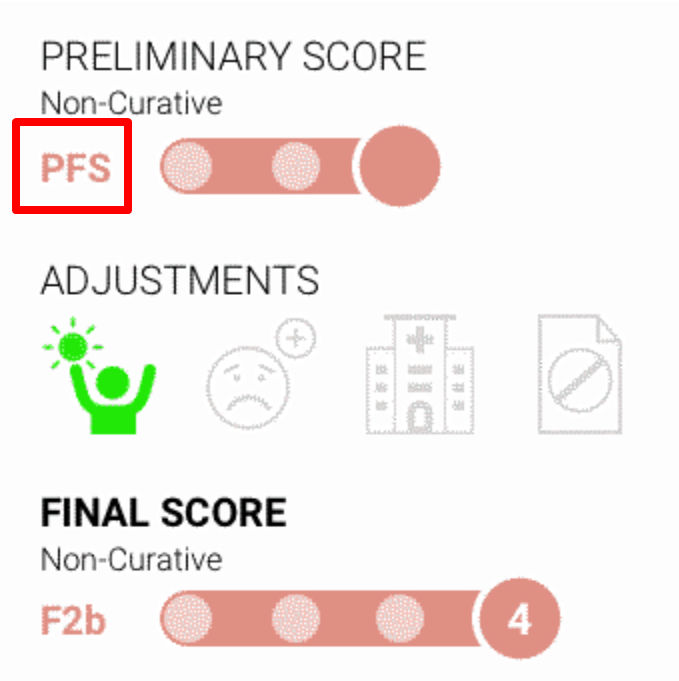

Detailed Version

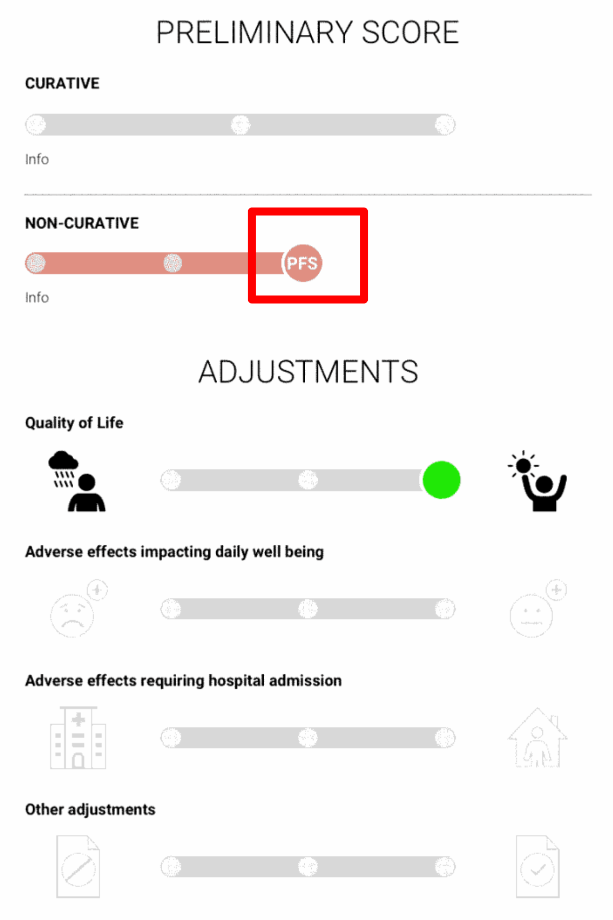

- The adjustments panel:
  1. QoL: Quality of life improved or worsened.
  2. Side effects that impact daily well being.
  3. Incremental toxicities that result in hospitalization (Form 2b).
  4. Other adjustments:
     - Plateau (Form 2a)
     - OS and PFS benefit (Form 2b)
     - PFS Plateau (Form 2b)
     - Early crossover (Form 2b).
     - Phase 4 experience (Form 3).

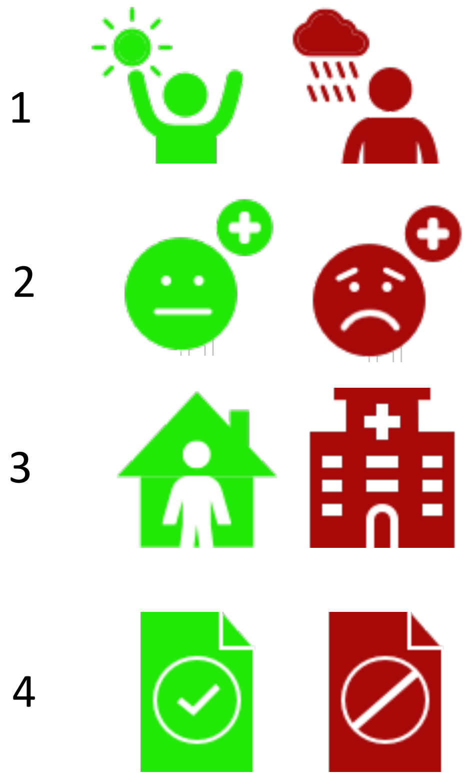

- The adjustments panel:
  - Positive adjustments are coded in green.
  - Negative adjustments are coded in red.

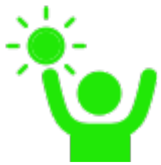

Upgrades the score.

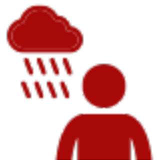

Downgrades the score

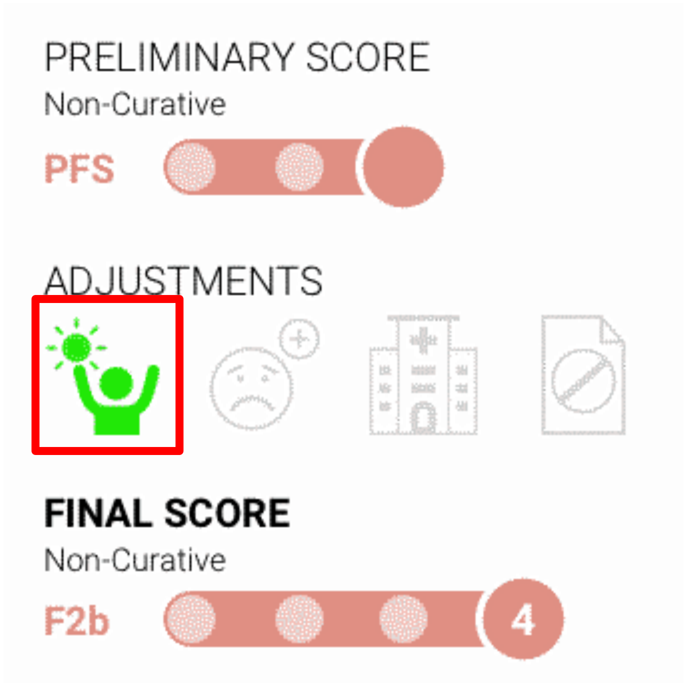

- The adjustments panel:
  - Some data about side effects and quality of life is measured but does not always influence the score.
  - Adjustments that do not result in an upgrade or downgrade to the score are depicted outlined.

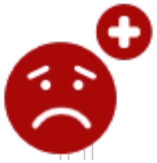

Influences the score (-1)

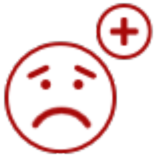

Does not influence the score.

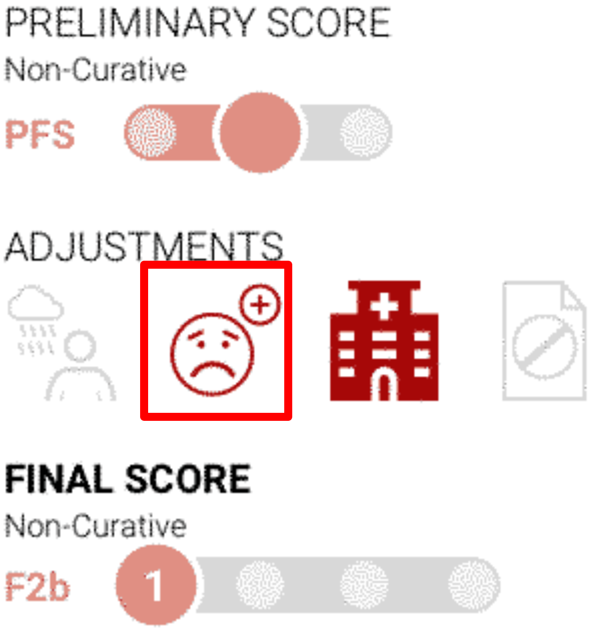

Detailed Version

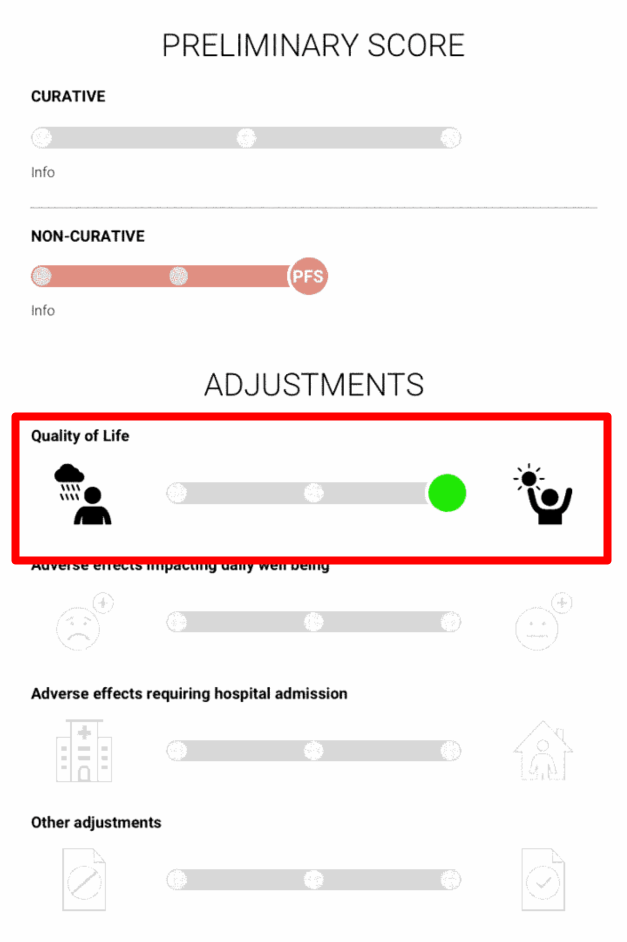

Detailed version

- The adjustments panel:
  1. Score Downgrade.
  2. Score Upgrade.
  3. Adjustment data pending.
  4. Same amount as control arm.
  5. Same amount and results in a downgrade.

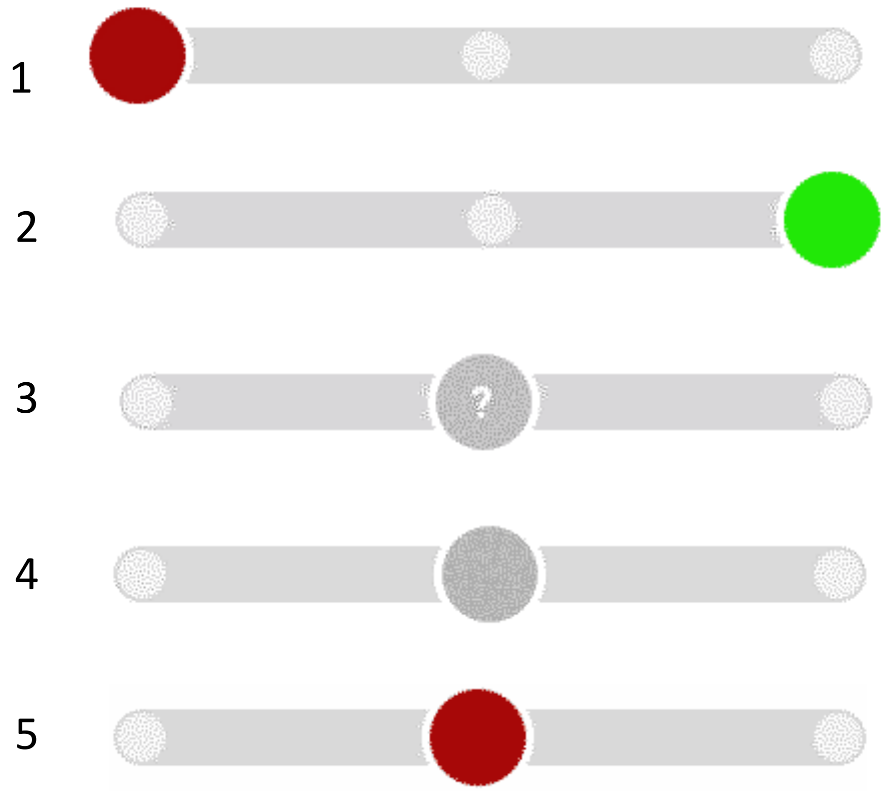

Example: CheckMate 141 (All patients)

- Reached a score of 4 *
  - *(All patients group)
- We can see that it is a therapy without curative intent that is a substantial improvement.
  - *It improves OS, QoL and side effects that impact daily well being.*
  - *It may be scored as a curative therapy in the future.*

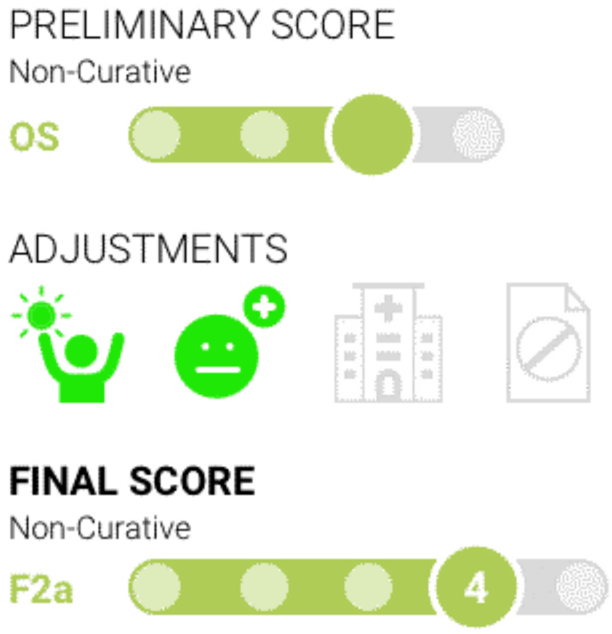

Example: CheckMate 141 (All patients)

PRELIMINARY SCORE

CURATIVE

Info

NON-CURATIVE

OS

Info

ADJUSTMENTS

Quality of Life

Adverse effects impacting daily well being

Adverse effects requiring hospital admission

Other adjustments

?

FINAL SCORE

CURATIVE

? ? ?

Overall Survival (OS) / Disease-Free Survival (DFS) / Pathologic Complete Response (pCR)

NON-CURATIVE

4

Overall Survival (OS)

Progression-Free Survival (PFS)

Quality of Life (QoL) / Relative Risk (RR) / Adverse Effects (AE)

Overall Response Rate (ORR) / Progression-Free Survival (PFS)

INFORMATION

Lorem ipsum: dolor sit amet, consectetur adipiscing elit, sed do eiusmod tempor incididunt ut labore et dolore magna aliqua. Ut enim ad minim veniam, quis nostrud exercitation ullamco laboris nisi ut aliquip ex ea commodo consequat. Duis aute irure dolor in reprehenderit in voluptate velit esse cillum dolore eu fugiat nulla pariatur. Excepteur sint occaecat cupidatat non proident, sunt in culpa qui officia deserunt mollit anim id est laborum.

Example: CheckMate 141 (All patients)

- Reached a score of 4 *
  - *(All patients group)
- The detailed version depicts that a quality metric adjustment could be achieved in the future (OS plateau).
- If achieved the clinical trial will be scored using the form for curative intent (Form 1).

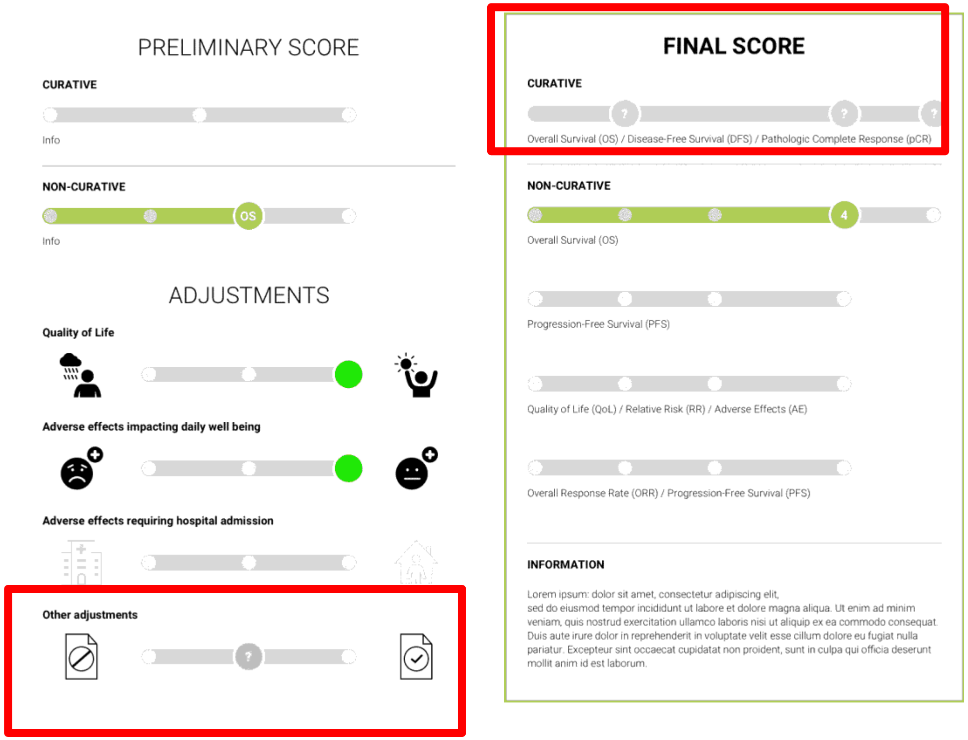

Example: PALOMA-3

- Reached a score of 4
- We can see that it is a therapy without curative intent that is a substantial improvement.
  - *It improves PFS and QoL.*
  - *It has OS data pending, therefore its score or form may change in the future.*

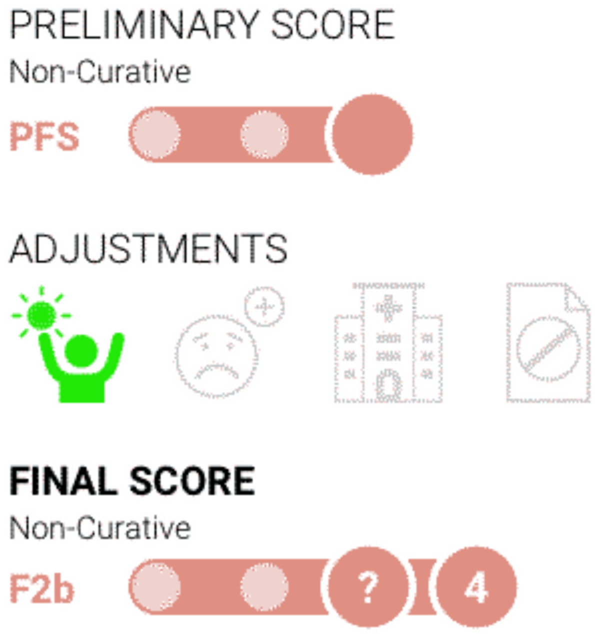

Example: PALOMA-3

PRELIMINARY SCORE

CURATIVE

Info

NON-CURATIVE

PFS

Info

ADJUSTMENTS

Quality of Life

Adverse effects impacting daily well being

Adverse effects requiring hospital admission

Other adjustments

?

FINAL SCORE

CURATIVE

Overall Survival (OS) / Disease-Free Survival (DFS) / Pathologic Complete Response (pCR)

NON-CURATIVE

? ? ? ? ?

Overall Survival (OS)

? 4

Progression-Free Survival (PFS)

Quality of Life (QoL) / Relative Risk (RR) / Adverse Effects (AE)

Overall Response Rate (ORR) / Progression-Free Survival (PFS)

INFORMATION

Lorem ipsum: dolor sit amet, consectetur adipiscing elit,
sed do eiusmod tempor incididunt ut labore et dolore magna aliqua. Ut enim ad minim veniam, quis nostrud exercitation ullamco laboris nisi ut aliquip ex ea commodo consequat. Duis aute irure dolor in reprehenderit in voluptate velit esse cillum dolore eu fugiat nulla pariatur. Excepteur sint occaecat cupidatat non proident, sunt in culpa qui officia deserunt mollit anim id est laborum.

Example: PALOMA-3

- Reached a score of 4 *
- The detailed version depicts that a quality metric adjustment could be achieved in the future (OS benefit).
- If achieved the clinical trial will be scored using the form for OS (Form 2a).
- If not achieved the score will be downgraded to a 3.

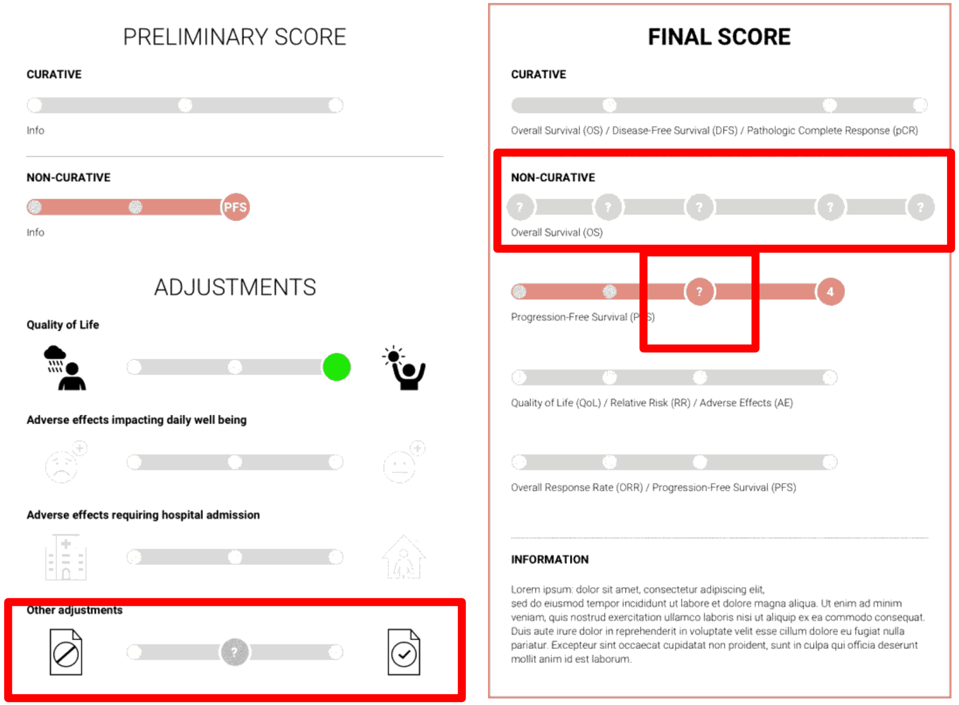

- Both of the previous examples would have seem to be similar without the visualisation.
  - *Both reached a score of 4 (substantial benefit).*

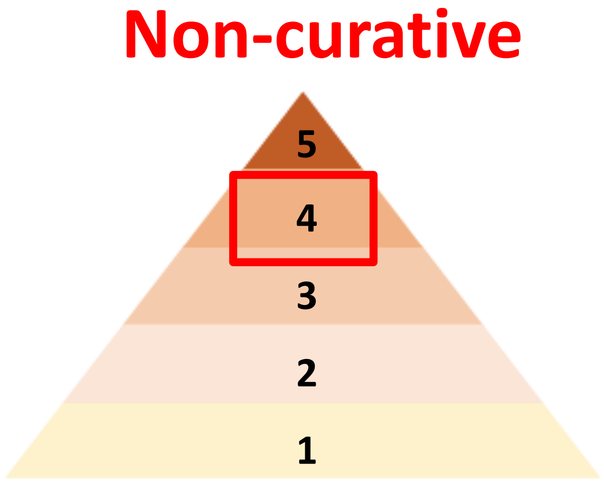

Thank you for reading the tutorial

- Take this short survey to give us feedback on the understandability of this visual tool:
  - Link
- Explore the catalogue of visualisations:
  - Link
- Send us a message:
  - mcbs@esmo.org
