# Supplementary material for: The ESMO-Magnitude of Clinical Benefit Scale (ESMO-MCBS) visualisation: picturing the evidence of clinical benefit of clinical trial data
Source: ESMO Real World Data Digit Oncol. 2025 Aug 26;9:100171. doi: 10.1016/j.esmorw.2025.100171 (PMC12836693; doi:10.1016/j.esmorw.2025.100171)
Supplement: Supplementary Material 4 [file mmc4.docx]

 
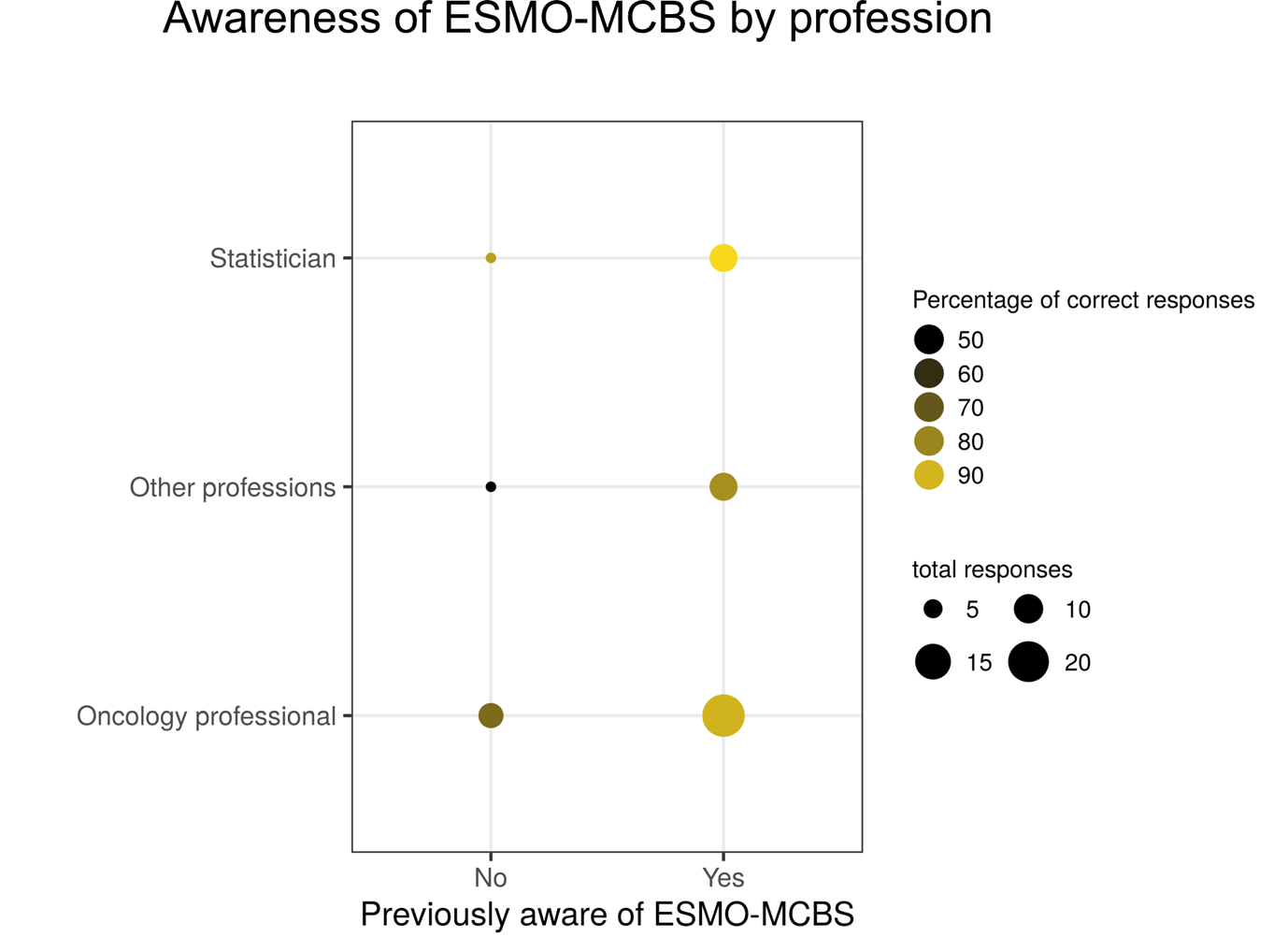


**Supplementary Figure 1:** **Percentages of correct answers for different profession subsets**

Bubble plot depicting the amount and percentage of correct responses for the survey sections evaluating the understanding of the ESMO-MCBS visualisation. Subsets are depicted for aggregates of professions reported in question two of the survey. Statisticians: Statistician, Biostatistician. Oncology professionals: Medical oncologist, Medical oncology fellows, Oncology pharmacist, Pharma representative. Other professions: PhD, Molecular biologist, Geneticist, Patient/Patient advocate, information technology (IT), Journalist. Subsets are further separated into two groups that reported having or not having previous knowledge of the ESMO-MCBS.
